# Clonally expanded HIV-1 proviruses with 5**′**-leader defects can give rise to nonsuppressible residual viremia

**DOI:** 10.1172/JCI165245

**Published:** 2023-03-15

**Authors:** Jennifer A. White, Fengting Wu, Saif Yasin, Milica Moskovljevic, Joseph Varriale, Filippo Dragoni, Angelica Camilo-Contreras, Jiayi Duan, Mei Y. Zheng, Ndeh F. Tadzong, Heer B. Patel, Jeanelle Mae C. Quiambao, Kyle Rhodehouse, Hao Zhang, Jun Lai, Subul A. Beg, Michael Delannoy, Christin Kilcrease, Christopher J. Hoffmann, Sébastien Poulin, Frédéric Chano, Cécile Tremblay, Jerald Cherian, Patricia Barditch-Crovo, Natasha Chida, Richard D. Moore, Michael F. Summers, Robert F. Siliciano, Janet D. Siliciano, Francesco R. Simonetti

**Affiliations:** 1 Department of Medicine, Johns Hopkins University School of Medicine, Baltimore, Maryland, USA.; 2 Department of Chemistry and Biochemistry, University of Maryland, Baltimore County, Baltimore, Maryland, USA.; 3 Department of Molecular Microbiology and Immunology, Johns Hopkins Bloomberg School of Public Health, Baltimore, Maryland, USA.; 4 Institute for Basic Biomedical Sciences, Johns Hopkins University School of Medicine, Baltimore, Maryland, USA.; 5 Clinique L’Agora, Montreal, Canada.; 6 Centre de Recherche du Centre Hospitalier de l’Université de Montréal (CHUM), Montreal, Canada.; 7 Département de Microbiologie, Immunologie et Infectiologie, Université de Montréal, Montreal, Canada.; 8 Howard Hughes Medical Institute, Baltimore, Maryland, USA.

**Keywords:** AIDS/HIV, T cells

## Abstract

**Background:**

Antiretroviral therapy (ART) halts HIV-1 replication, decreasing viremia to below the detection limit of clinical assays. However, some individuals experience persistent nonsuppressible viremia (NSV) originating from CD4^+^ T cell clones carrying infectious proviruses. Defective proviruses represent over 90% of all proviruses persisting during ART and can express viral genes, but whether they can cause NSV and complicate ART management is unknown.

**Methods:**

We undertook an in-depth characterization of proviruses causing NSV in 4 study participants with optimal adherence and no drug resistance. We investigated the impact of the observed defects on 5′-leader RNA properties, virus infectivity, and gene expression. Integration-site specific assays were used to track these proviruses over time and among cell subsets.

**Results:**

Clones carrying proviruses with 5′-leader defects can cause persistent NSV up to approximately 10^3^ copies/mL. These proviruses had small, often identical deletions or point mutations involving the major splicing donor (MSD) site and showed partially reduced RNA dimerization and nucleocapsid binding. Nevertheless, they were inducible and produced noninfectious virions containing viral RNA, but lacking envelope.

**Conclusion:**

These findings show that proviruses with 5′-leader defects in CD4^+^ T cell clones can give rise to NSV, affecting clinical care. Sequencing of the 5′-leader can help in understanding failure to completely suppress viremia.

**Funding:**

Office of the NIH Director and National Institute of Dental and Craniofacial Research, NIH; Howard Hughes Medical Institute; Johns Hopkins University Center for AIDS Research; National Institute for Allergy and Infectious Diseases (NIAID), NIH, to the PAVE, BEAT-HIV, and DARE Martin Delaney collaboratories.

## Graphical abstract

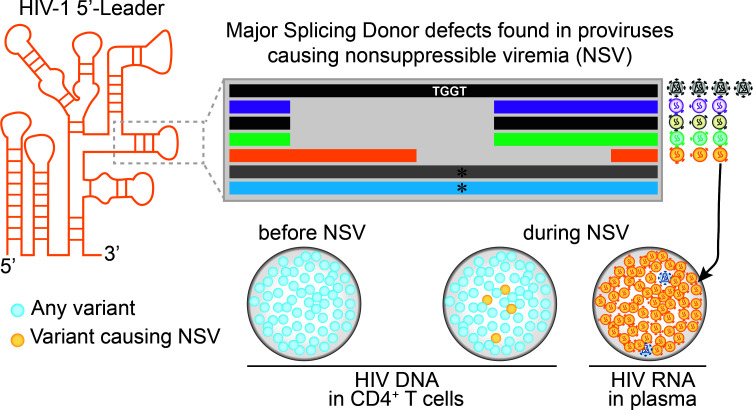

## Introduction

Treatment with antiretroviral therapy (ART) rapidly reduces plasma HIV-1 to below the detection limit of clinical assays, prevents infection of new cells, and dramatically reduces HIV-1–associated morbidity and mortality (1). However, ART is not curative because the virus persists in a stable latent reservoir in resting memory CD4^+^ T cells, necessitating life-long adherence to ART (2–5).

A small fraction of reservoir cells become activated daily and produce trace levels of free virus (1–3 copies/mL) detectable with ultrasensitive assays (6, 7). These viruses are nonevolving archival sequences sensitive to the current regimen (8–10), indicating that residual viremia reflects virus release from stable reservoirs rather than ongoing replication cycles. Residual viremia cannot be suppressed by treatment intensification (11, 12) and is often dominated by identical sequences reflecting extensive proliferation of infected T cells (10, 13–15), which contributes to reservoir stability (16–20).

Some treated people living with HIV-1 (PLWH) experience sustained periods of detectable viremia, prompting reassessment of adherence, drug resistance, and drug concentrations. Regimen optimization or intensification often fails to suppress viremia (11, 12, 21). Recent studies have shown that expanded T cell clones carrying infectious proviruses can cause nonsuppressible viremia (NSV) (14, 15).

In treated PLWH, more than 90% of proviruses are defective (22, 23) with large deletions and/or APOBEC3G/F-induced G-to-A hypermutations (24). Some defective proviruses can express HIV-1 RNA and produce viral proteins (25–30). A recent study suggested that a fraction of viruses found in plasma before ART was defective due to small deletions and frameshifts (31). Some defective proviruses have small deletions in the 5′-leader (5′-L) upstream of *gag* (23, 32, 33). This region contains regulatory elements orchestrating genomic RNA dimerization and packaging, reverse transcription, proviral gene expression, and RNA splicing and translation (34–39). Proviruses with small 5′-L deletions make up 5% to 10% of proviral populations, are found in most PLWH, often in expanded clones (23, 32, 40–42), and may produce viral mRNA and p24 protein (26, 33). Given the potential of 5′-L defects to affect multiple steps in the viral cycle, these proviruses would not be expected to cause NSV or viral rebound. However, we demonstrate that these proviruses are a common cause of NSV that complicates clinical management.

## Results

### Emergence of NSV despite ART.

**Figure 1:**
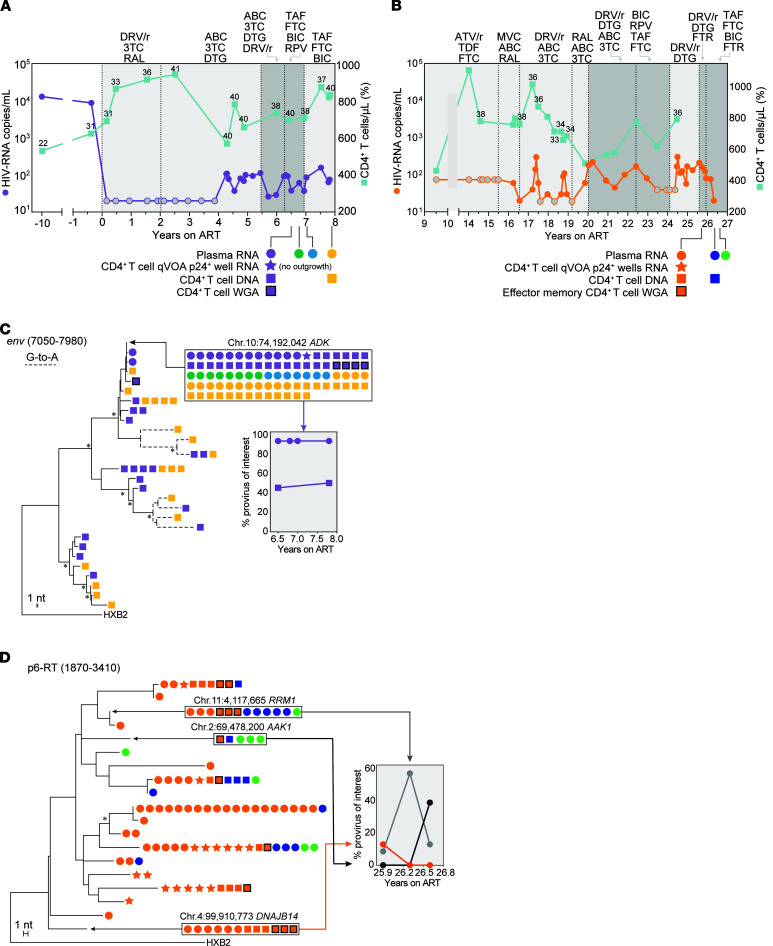
Clinical history of 2 study participants with NSV and analysis of HIV-1 populations in plasma and CD4^+^ T cells. (**A** and **B**) Plasma HIV-1 RNA and CD4^+^ T cell counts over time for P1 and P2. Gray circles indicate values below the limit of quantification. Numbers above squares indicate CD4^+^ T cell percentages. Light gray areas indicate standard ART. Dark gray areas indicate ART intensification. (**C**) Maximum likelihood tree analysis of *env* single-genome sequences from P1. Dashed branches indicate sequences with hypermutation. Tree nodes with bootstrap values above 80 are marked by asterisks. Identical sequences matching proviruses with integration and full genome data are highlighted in boxes. Chromosomal location is indicated above boxed area. Frequencies of variants of interests over time are shown in the graph insert. (**D**) Maximum likelihood tree analysis of P6-RT single-genome sequences from P2. Only plasma and viral outgrowth RNA sequences are shown, together with matching proviral DNA sequences (the complete tree is shown in Supplemental Figure 3). 3TC, lamivudine; ABC, abacavir; FTC, emtricitabine; TDF, tenofovir disoproxil fumarate; TAF, tenofovir alanfenamide; DRV/r, darunavir-ritonavir; ATV/r, atazanavir-ritonavir; RAL, raltegravir; DTG, dolutegravir; BIC, bictegravir; MVC, maraviroc; RPV, rilpivirine; FTR, fostemsavir.

Participants were referred for persistently detectable plasma viremia despite optimal adherence. Their characteristics and HIV-1 reservoir measurements are summarized in Supplemental Table 1, Figure 1, and Supplemental Figures 1 and 2 (supplemental material available online with this article; https://doi.org/10.1172/JCI165245DS1). The 4 participants had been living with HIV-1 for more than 15 years (range, 15–32 years) and had been on long-term ART (range, 7.8–27 years) to which they responded with peripheral CD4^+^ T cell recovery and undetectable viral loads. After years of viral suppression (<20 copies/mL), they experienced detectable viremia not explained by changes in adherence, drug bioavailability, or resistance. Treatment optimization, intensification, or deintensification had no effect (Figure 1, A and B, and Supplemental Figures 1 and 2). Participant 1 (P1) had persistently detectable viremia for 4.3 years, with a median of 80 HIV-1 RNA copies/mL of plasma (range, 37–156 copies/mL). P2 experienced intermittent periods of detectable viremia for more than 10 years, with a median of 75 copies/mL (range, <20–300 copies/mL). P3 had persistently detectable viremia for 4.6 years, with a median of 123 HIV-1 RNA copies/mL (range, 26–857 copies/mL) (Supplemental Figure 1). P4 represents an extreme example of NSV, maintaining a median of 2,979 copies/mL (range, 1,145–5,138 copies/mL) for almost 2 years (Supplemental Figure 2). Clinical histories are summarized in Supplemental Results.

### Clonal origin of HIV-1 viremia.

To investigate the cause of NSV, we recovered longitudinal single-genome sequences of plasma RNA (Figure 1 and Supplemental Figures 1, 2, and 3). In P1, 44 plasma envelope (*env*) sequences were identical, except for 2 sequences with 1 nt difference each, likely reflecting PCR error (43). The plasma sequences at all 4 time points spanning 1.5 years were identical, suggesting a predominant plasma clone resulting from cell proliferation and proviral expression (10, 14, 15). Analyses of U5-*gag* and p6-RT sequences confirmed that viremia was due to a single variant with no resistance mutations (Supplemental Figure 3, A and B). *Env* sequences in plasma at the time of ART initiation (in 2013) were diverse and belonged to 2 distinct lineages (Supplemental Figure 3C). The provirus causing NSV on ART fell immediately outside 1 of these 2 main clades and showed no significant divergence from pre-ART sequences (Supplemental Figure 3C). Proviruses from 6.5 and 7.8 years on ART (Figure 1C) were diverse, but showed no increase in diversity (4.2% and 3.6%, respectively) or genetic shift (test for panmixia, *P =* 0.52) over time. Most importantly, the sequences of 1 proviral variant were identical to the plasma sequences and represented approximately 50% of proviral sequences at each time point (17/35 out of 35 and 21/39 out of 39, respectively).

In P2, multiple variants contributed to viremia (Figure 1D and Supplemental Figure 3), likely reflecting a larger reservoir as indicated by intact proviral DNA assay (IPDA) and quantitative viral outgrowth assays (QVOAs) (Supplemental Table 1). From the first time point, we obtained 48 p6-RT RNA sequences belonging to 10 distinct variants that represented between 2.1% and 41.7% of virus in plasma. HIV-1 viremia decreased at later time points, resulting in lower sampling depth (11 and 8 sequences, respectively). Across 3 time points, we observed a variable representation of HIV-1 variants in plasma, and only 5 variants were found more than once, likely due to lower sampling and the variable virus production from infected clones over time (44). Three variants matched sequences recovered from viral outgrowth assays, representing replication-competent proviruses contributing to NSV (15). Interestingly, the most abundant plasma variant in plasma at the first time point (41.7%) did not match any of the 142 proviral sequences or the 16 outgrowth sequences from QVOA (Supplemental Figure 3). This observation is not uncommon, as infected cells from this clone could be at very low frequency in blood or are tissue-resident cells (45). For P3, 27/28 plasma p6-RT sequences represented a single variant (Supplemental Figure 1B) that was rare among infected cells (2.7% ± 5.2%, Supplemental Figure 1, B and C). For P4, 20/20 plasma p6-RT RNA sequences from a sample collected at 26.5 years on ART were identical, drug sensitive, and matched 4 RNA sequences obtained previously with a clinical HIV-1 genotype assay (Supplemental Figure 2). Proviral sequences (*n =* 35) were diverse and none matched the plasma variant, reflecting the low frequency of the provirus causing viremia (less than 2.8% ± 5.4%) (Supplemental Figure 2B). Of note, we did not detect drug resistance in any of the sequences recovered from the 4 participants. Together, these results show that NSV often comprises identical sequences despite enormous proviral diversity.

### NSV can arise from proviruses with 5′-L defects.

To investigate replication competence of the proviruses causing NSV, we performed QVOAs (46). In P1, despite the input of 11.12 million CD4^+^ T cells, there was no exponential outgrowth after 28 days (<0.06 IUPM, Supplemental Figure 4). One well showed a borderline p24 signal (~3 pg/mL) on day 21. Single-genome U5-*gag* sequences from cell-associated RNA and supernatant virions (Supplemental Figure 4) were identical to those of plasma virus, suggesting in vitro virus production without exponential replication of the provirus causing NSV.

The QVOA from P2 revealed a large reservoir size (15 IUPM) allowing recovery of 6 unique variants from 11/24 p24^+^ wells. The most abundant QVOA variant represented 37% of positive wells and matched 1 plasma clone found at all time points (Figure 1D). Two additional outgrowth viruses matched other clonal plasma sequences. Other plasma viruses from P2 were not detected in the QVOA. QVOAs were not performed for P3 and P4 due to sample availability.

**Figure 2:**
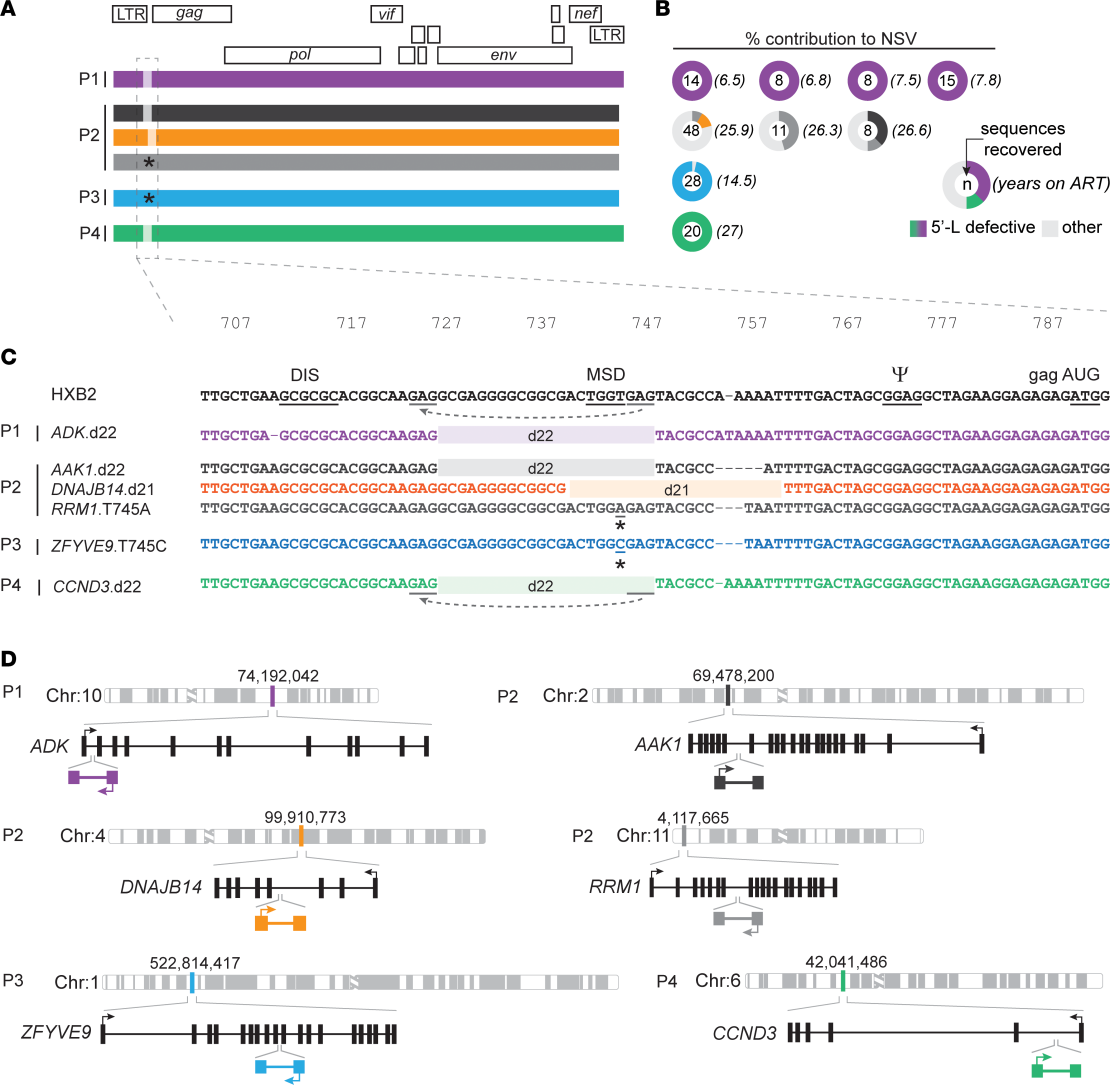
Paired full-genome sequencing and integration site analysis of 5′-L–defective proviruses causing NSV. (**A**) Mapped sequences of proviruses contributing to plasma HIV-1 RNA in each participant. Proviruses of interest are color-coded throughout the figure. Light-shaded areas indicate small 5′-L deletions. Stars indicate point mutations affecting the MSD site. (**B**) Percentage contributions to NSV of defective proviruses of interest. (**C**) 5′-L defects aligned to the HXB2 reference. DIS, dimerization initiation signal; PSI, packaging signal; AUG, Gag start codon. Dashes indicate length polymorphisms. Gray lines highlight GAG repeats at deletion junctions causing misplaced jumping of reverse transcriptase (dashed gray arrows). (**D**) Chromosomal and gene locations of proviruses causing viremia. Sets of arrows indicate direction of proviral and host gene transcription. Schematic gene tracks are shown in black, with vertical bars representing exons.

To explore the replication competence of plasma clones not detected in the QVOA, we performed whole-genome amplification of CD4^+^ T cell DNA (Figure 2, A and B). Near full-length proviral sequencing revealed subtype B, R-tropic, drug-sensitive proviruses (Supplemental Figure 2D) with intact open reading frames. However, they all showed defects in the 5′-L (Figure 2C). Surprisingly, proviruses causing NSV from P1, P2, and P4 shared the same 22 nt deletion (HXB2 positions 727–748) affecting the dimerization hairpin and the major splicing donor (MSD) site (TGGT) (Figure 2B). The appearance of the same deletion in different individuals is likely favored by short repeats (GAG) at the deletion junction (Figure 2B), consistent with microhomology-driven template switching during minus-strand synthesis (47). Two more proviruses causing NSV in P2 had 5′-L defects: a 21 nt deletion (HXB2 positions 740–760) and a T-to-A mutation in position 745, both affecting the MSD site. Similarly, in P3 we found that 100% of plasma virus had an MSD mutation, also at position 745 (T-to-C) (Supplemental Figure 1C). We confirmed the clonal nature of these recurring defective proviruses using integration site analysis (Figure 2D and Supplemental Table 2, and Supplemental Results). These 5′-L defective proviruses were labeled with the host gene symbol and the specific 5′-L defect (e.g., *ADK*.d22). These results indicate that otherwise intact, clonally expanded proviruses with 5′-L defects can cause sustained NSV.

### Defective 5′-Ls exhibit modest changes in dimerization and nucleocapsid binding.

**Figure 3:**
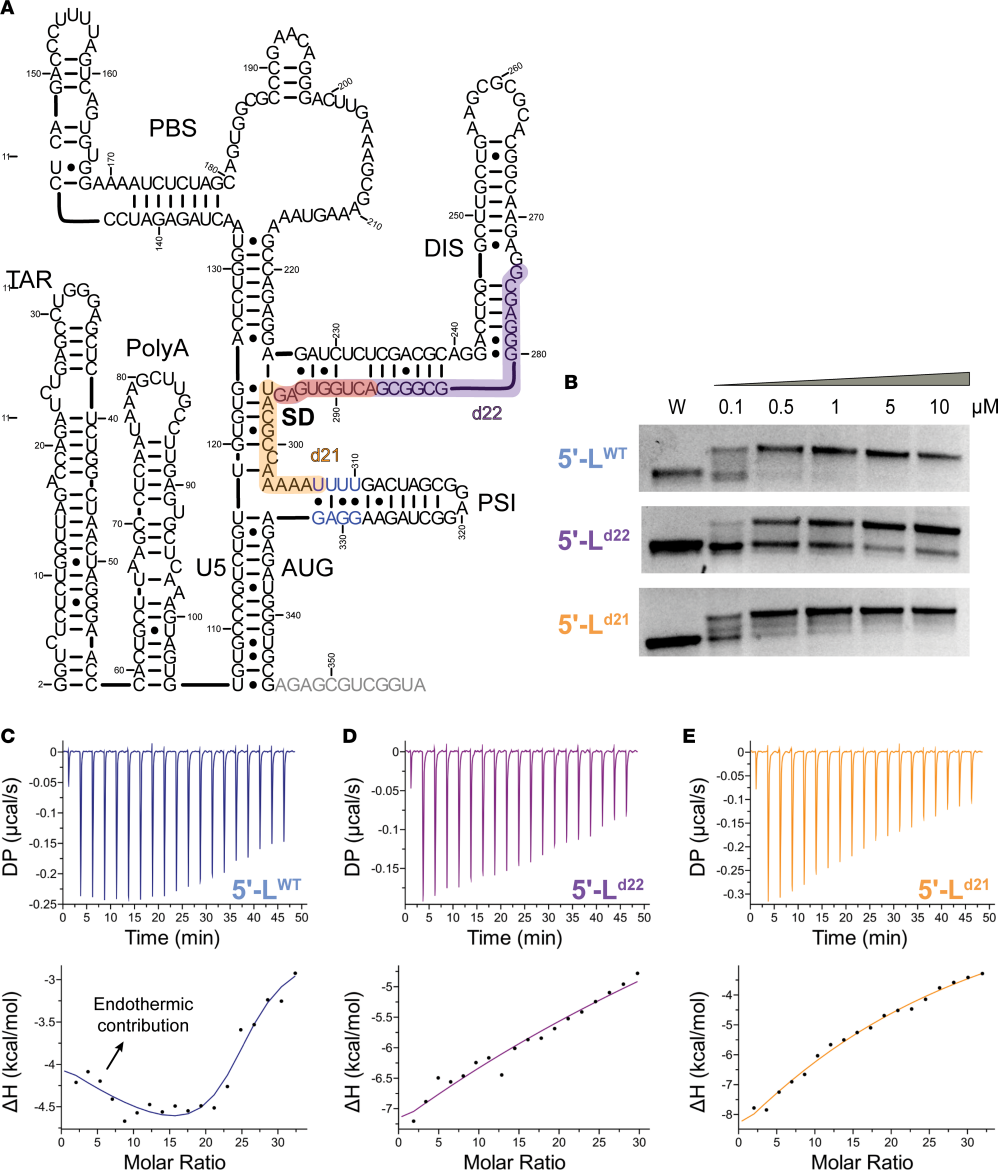
5′-L deletions alter dimerization propensities and NC-binding properties. (**A**) Secondary structure of the HIV-1 NL4-3 gRNA 5′-L with patient deletions indicated in purple (d22) and orange (d21), with a region of overlap within the splice donor (SD) shown in red. High-affinity binding sites with endothermic contribution on ITC binding within the psi hairpin are indicated with blue text. Gray text indicates the portion of AUG truncated to better study the dimer and its initial binding sites by ITC. (**B**) Concentration-dependent dimerization assays of the full leader show that the WT and d21 constructs maintain similar dimerization propensities, while the d22 variants exhibit reduced dimerization. (**C**–**E**) ITC isotherms for the truncated dimeric 5**′**-L titrated with low protein-to-RNA ratios. WT exhibits previously described initial binding with an endothermic contribution (**C**) that is not seen for the d22 (**D**) or d21 (**E**) 5**′**-L constructs.

The 22 nt (5′-L^d22^) and 21 nt (5′-L^d21^) deletions remove the MSD site and portions of the dimer initiation site (DIS) and packaging (psi, ѱ) hairpins, respectively, and disrupt the tandem 3-way junction (Figure 3A) involved in genome packaging (37, 39) and in high-affinity binding to Gag (48). Therefore, we compared dimerization of 5′-L^d22^ and 5′-L^d21^ mutants to that of the HIV-1 NL4-3 (5′-L^WT^) using native agarose gels. Under physiologic ionic conditions, all 3 RNAs dimerized (Figure 3B), with the 5′-L^d22^ form showing a modest reduction in dimer stability (~5-fold higher *K_D_*), probably because the deletion occurs within the lower stem of the DIS hairpin. In contrast, the deletions in the 5′-L^d21^ RNA reside outside the DIS hairpin element and do not appear to influence RNA dimerization (Figure 3B).

To determine whether the deletions affect Gag binding, we used isothermal titration calorimetry (ITC). The 5′-L^WT^ RNA bound approximately 32 nucleocapsid (NC) molecules, whereas the 5′-L^d21^ and 5′-L^d22^ bound 20 and 25 NC proteins, respectively (Supplemental Figure 5 and Supplemental Table 3). To probe for NC-induced RNA unwinding associated with the initial, highest affinity NC binding sites, we conducted additional ITC titrations at lower NC-to-RNA ratios (37). ITC titration profiles for 5′-L^WT^ exhibited a characteristic endothermic contribution to NC binding (Figure 3C) (37), but profiles for both mutants lack this feature (Figure 3, D and E). Prior work with fragments of the native 5′-L indicate that this endothermic term corresponds to approximately 4 highest affinity NC-binding sites (*K_D_*= ~40 nM) and is found to be essential for efficient RNA packaging (37). Taken together, these experiments show that 5′-L deletions found in proviruses causing NSV produce only modest reductions in dimerization and NC binding, consistent with retained genomic RNA packaging.

### 5′-L–defective proviruses are inducible and show rescue of gene expression through alternative splicing.

**Figure 4:**
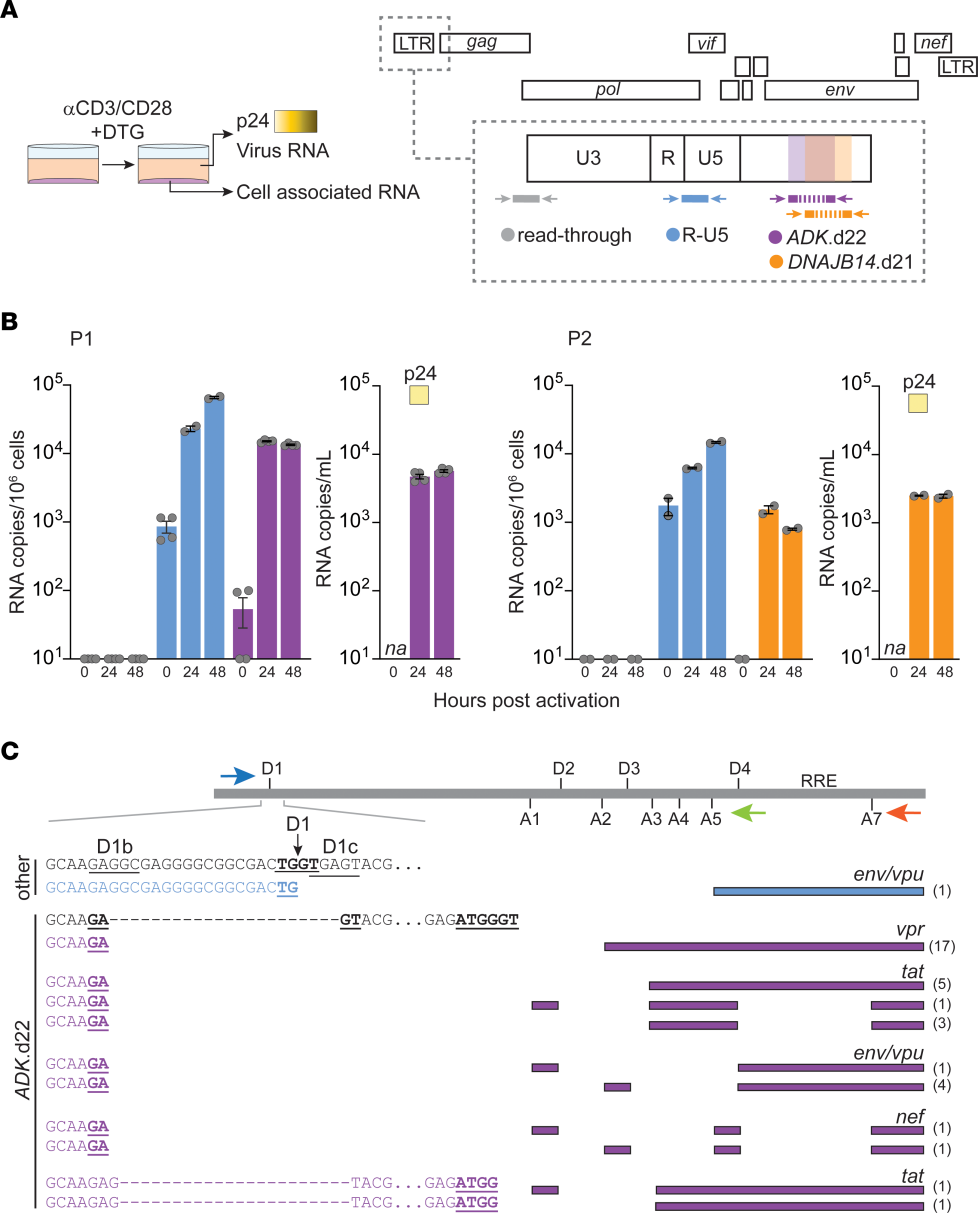
5′-L-defective proviruses are inducible and their genomic RNA is packaged and can use alternative splicing donors. (**A**) CD4^+^ T cells from P1 and P2 were cultured for 48 hours in the presence of dolutegravir (DTG) and anti-CD3/CD28 beads. Cells and supernatants were collected at 0, 24, and 48 hours. Right panel shows the location of primers and probes used to quantify viral RNA. Shaded areas indicate 5**′**-L deletion used to measure provirus-specific transcription. (**B**) Mean levels of read-through, total, and provirus-specific RNA detected in cells and supernatant upon T cell activation. Gray circles represent digital PCR replicate reactions. Error bars indicate SEM. (**C**) Singly and multiply spliced transcripts amplified at limiting dilution from cells at 48 hours. Arrows indicate primer locations. Ticks indicate the locations of splicing donors and acceptors in the HIV-1 genome. Major and alternative splicing donor sites are labeled in black. Mapped splicing junctions are underlined and in bold; 22 nt deletion is represented by dashed lines. Numbers in parentheses indicate the number of sequences recovered for each type of spliced variant.

To investigate the inducibility of proviruses responsible for NSV, we stimulated CD4^+^ T cells from P1 and P2 with anti-CD3/anti-CD28 beads and quantified cell-associated HIV-1 RNA (R-U5) at 0, 24, and 48 hours after stimulation (Figure 4). In addition, to selectively measure transcripts from proviruses of interest (*ADK*.d22 and *DNAJB14*.d21 from P1 and P2, respectively), we designed assays that would amplify only unspliced RNAs with those deletions (Figure 4A and Supplemental Figure 6). Finally, to rule out readthrough transcripts (49), we used an amplicon spanning the host gene–U3 junction. Upon stimulation, we observed a marked increase in total R-U5 RNA and in transcripts originating from the *ADK*.d22 and *DNAJB14*.d21 proviruses, which at 48 hours represented 20% and 5% of the total viral RNA, respectively. Of note, we detected *ADK*.d22 RNA at baseline, consistent with low-level spontaneous HIV-1 expression from this clone in peripheral blood cells. We did not detect chimeric RNA at the host gene–LTR junction. This was expected, given the opposite transcriptional orientation of the proviruses relative to the surrounding host genes and suggests that provirus-specific transcripts are LTR driven. We also detected low levels of p24 in culture supernatant and provirus-specific virion-associated RNA at 24 and 48 hours (Figure 4B), supporting the conclusion that genomic RNA from these defective proviruses can be packaged.

Given the absence of the MSD site due to the 22 nt deletion, we analyzed splicing in cells carrying the *ADK*.d22 provirus. CD4^+^ T cells from P1 were activated for 48 hours with anti-CD3/CD28 beads, and HIV-1 cDNA was synthesized from cell-associated RNA using primers annealing immediately downstream of 2 major splicing acceptors, A5 and A7. Single genome amplification of singly and multiply spliced HIV-1 RNA revealed an alternative noncanonical splicing donor site, AGA*GT, created by the 22 nt deletion in the ADK provirus. This deletion results in the fusion of 2 alternative splice donor sites, D1b and D1c (Figure 4C). Of 36 mRNA sequences, 35 were derived from the *ADK*.d22 provirus, and 33 of these used the alternative splice donor. The remaining 2 sequences used a previously described alternative splice donor, ATGG*GT, at the *gag* gene translation start site (26). We detected mRNAs for Env, Vpu, Tat, and Nef, but not for Rev, which is required for the efficient export of intron-containing RNAs, and for Vif, which, however, represents only approximately 1% of all mRNA species of the 4 kb class (50–52). Together, these results show that proviruses with an MSD deletion can be induced and can express some viral genes using an alternative strong donor site.

### Small 5-′L deletions result in noninfectious viral particles with decreased env incorporation.

**Figure 5:**
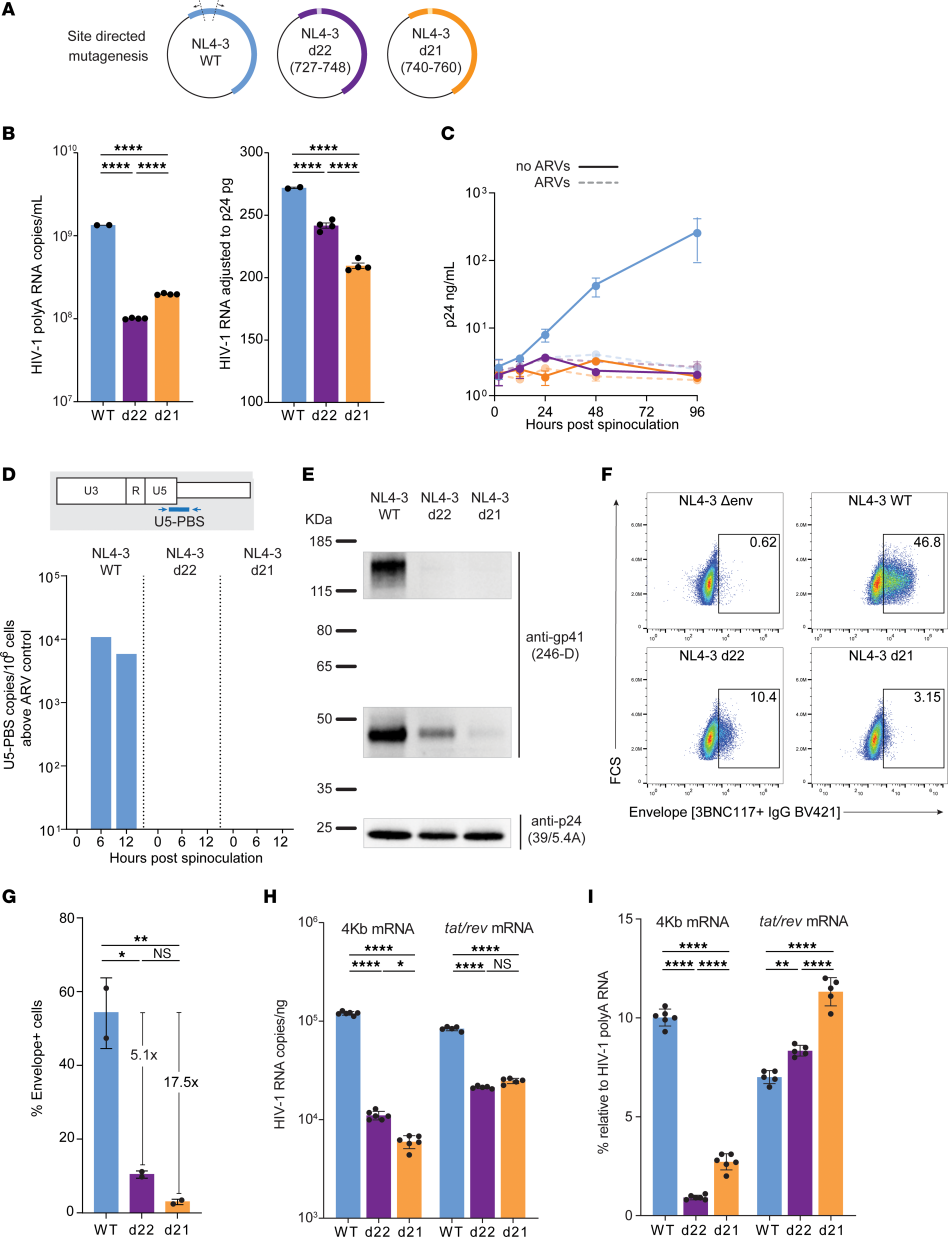
5′-L deletions lead to noninfectious particles lacking env incorporation. (**A**) Deletions found in proviruses causing viremia were introduced in an NL4-3 expression plasmid by site-directed mutagenesis. Deletion start and end positions relative to HXB2 are indicated in parentheses. (**B**) Copies of HIV-1 RNA recovered at 72 hours after transfection of 293T cells, expressed as copies per mL (left) or normalized by p24 pg/mL (right). (**C**) Spinoculation of primary CD4^+^ T cells shows exponential increase in p24 levels only with WT NL4-3, in the absence of antiretrovirals (ARVs: TDF, FTC, DTG). (**D**) Reverse transcription was assessed by measuring late cDNA products by ddPCR targeting the U5-PBS junction. Primary CD4^+^ T cells were collected at 0, 6, and 12 hours after spinoculation with and without ARVs. U5-PBS copies detected in the presence of ARVs, which are the result of incomplete DNAseI digestion of plasmid carryover from transfection, were subtracted from copies detected in conditions without ARVs. (**E**) Virus produced upon 293T transfection was pelleted by ultracentrifugation, lysed, normalized by p24, and used for Western blots with primary antibodies specific to p24 and gp41. (**F**) Surface staining of HIV-1 env on 293T cells 24 hours after transfection. (**G**) Frequency of env-positive cells transfected with WT versus 5**′**-L deletions. Fold reduction relative to WT is indicated above each mutant. Results from 2 transfection experiments are shown. Each circle represents the average of 2 technical replicates. (**H**) Quantification of cell-associated spliced HIV-1 transcripts belonging to the 4 kb class or *tat/rev* mRNA normalized to RNA ng. (**I**) Percentage of spliced transcripts relative to total HIV-1 polyA RNA. Error bars indicate SEM (**B**, **H** and **I**) or SD (**C** and **F**). Statistical significance between conditions was determined by 1-way ANOVA. *P < 0.05; **P <0.01, ***P <0.001.

To investigate the impact of the 5′-L defects on replicative fitness, we introduced the deletions observed into a reference proviral construct (NL4-3) and generated virions by transfection of 293T cells (Figure 5A). At 72 hours after transfection, virus production was reduced 10-fold relative to WT (Figure 5B). However, after normalization by p24, d22 and d21 had only a modest reduction in HIV-1 RNA packaging, as predicted by the 5′-L analyses described above (Figure 3).

To investigate replication capacity, we spinoculated activated primary CD4^+^ T cells with WT or 5′-L mutant viruses (Figure 5C). Only the WT virus showed an exponential increase in supernatant p24, while d22 and d21 showed persistently low p24 levels that were not affected by antiretrovirals and were likely the result of p24 carryover from the spinoculation (Figure 5C). Reductions in RNA dimerization can reduce replicative capacity by affecting reverse transcription efficiency (53–55). Therefore, we measured late products of reverse transcription containing the U5-PBS junction in primary CD4^+^ T cells. The d22 and d21 mutants showed no cDNA at 12 hours after infection (Figure 5D), suggesting a defect early in or upstream of reverse transcription. To determine whether the deletions would prevent the initiation of reverse transcription due to a disruption of primer binding site (PBS) secondary RNA structure, we performed an in vitro transfer RNA–binding (tRNA-binding) assay. Both WT and mutant 5′-L RNAs bound tRNA (Supplemental Figure 7), suggesting that the deletions did not affect PBS structure.

Based on these observations, we hypothesized that the deletions could prevent viral entry. After normalization by input p24, Western blots of pelleted d22 and d21 virions showed negligible gp160 and gp41 (Figure 5E), indicating that the loss of infectivity was due to insufficient env incorporation. To determine whether the lack of virion-associated Env is caused by reduced surface Env on infected cells, we studied Env on 293T cells 24 hours after transfection by flow cytometry (Figure 5F). Compared with WT, d22 and d21 showed 5.1- and 17.5-fold reductions in Env^+^ cells (WT, 54%; d22, 10.5%; d21, 3.1% Env^+^ cells, respectively) (Figure 5G). Moreover, mean fluorescence intensity was significantly lower, suggesting that Env expression was also reduced at the individual cell level (*P <* 0.0001). We hypothesized that the lower Env expression is caused by alterations in splicing due to the 5′-L deletions. Therefore, we measured cell-associated HIV-1 RNA with assays targeting all polyadenylated transcripts (56), 4 kb class of spliced mRNA including polycistronic *vpu/env* transcripts, and multiply spliced tat/rev mRNA (57). 5′-L deletions led to a significant reduction not only in absolute copies of the 4 kb class of spliced mRNA (Figure 5, H and I), but also in its relative percentage among all transcripts (WT, 10%; d22, 0.9%; d21, 2.7%; *P <* 0.0001). Conversely, the relative abundance of *tat/rev* mRNA was increased in the 5′-L mutants (Figure 5I). Finally, we imaged 293T cells by transmission electron microscopy to visualize viral particles at 24 hours after transfection (Supplemental Figure 8). While a large fraction of cells transfected with WT NL4-3 showed high virus production and underwent cell death, transfection with d22 and d21 led to low-level production of viral particles and little cytopathic effect. Of note, viral particles had an immature appearance, with the typical radial distribution of Gag polyproteins (58). Together, these results show that the 5′-L defects observed in the plasma clones from P1, P2, and P4 cause splicing defects that reduce but do not completely abrogate the production of mRNAs encoding Env, resulting in lower virion Env protein levels and noninfectious viral particles.

### Tracking cells carrying 5′-L defective proviruses over time.

**Figure 6:**
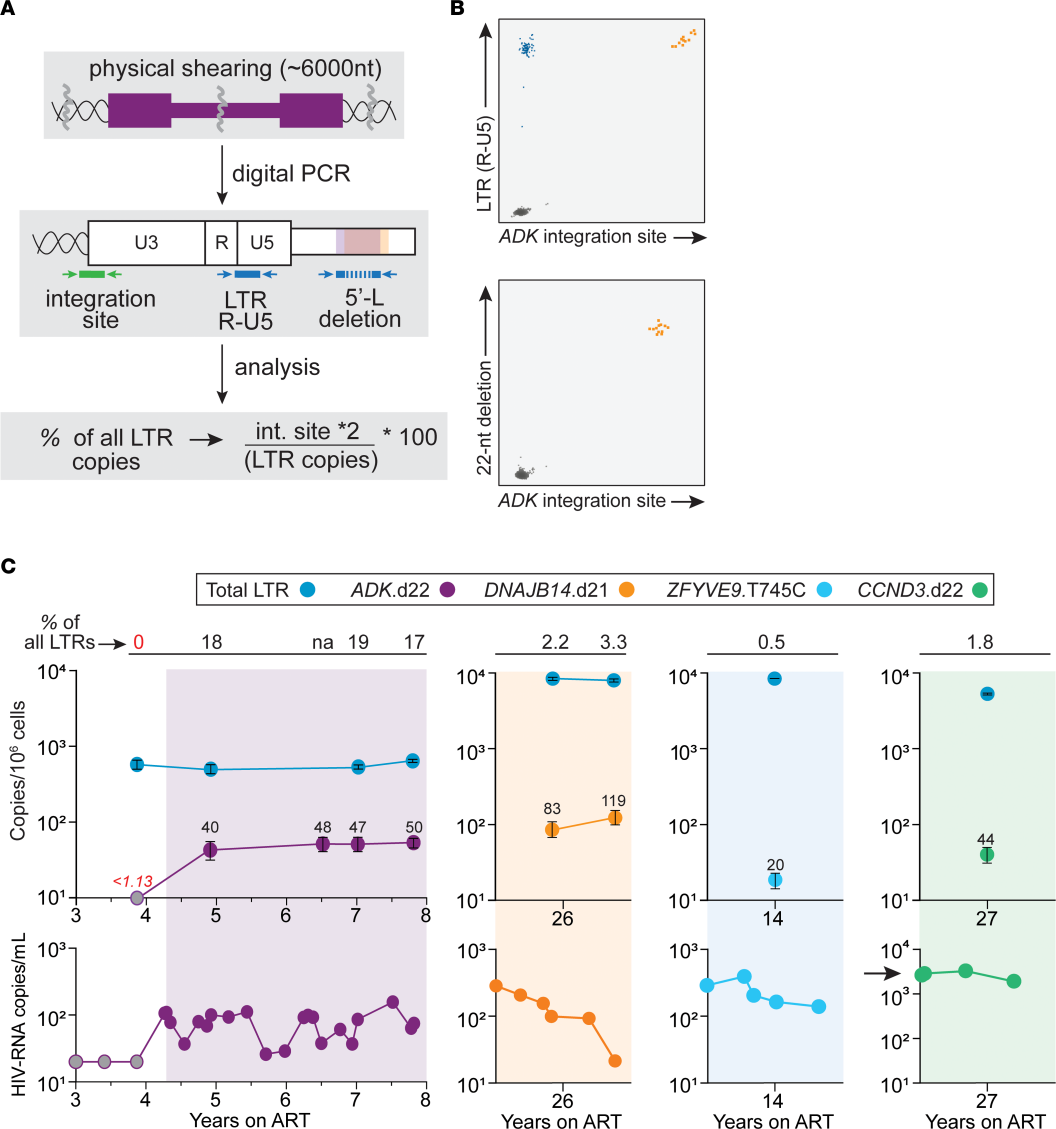
Proviruses that contribute to plasma virus are stable over time and show clonal expansion concurrent with onset of NSV. (**A**) Experimental approach: genomic DNA is sheared to obtain fragments shorter than 6000 nt. Total LTR copies are quantified with primers and probe targeting both R-U5 junctions, while proviruses of interest are quantified targeting the site of HIV-1 integration or the 5**′**-L deletion. (**B**) Representative ddPCR 2D plots of *ADK*.d22-specific assays. Due to the proximity of the integration site and 5**′** the R-U5 junction, most proviruses of interest are double positive. (**C**) Longitudinal quantification of total LTR copies and proviruses contributing to viremia. Numbers above symbols indicate mean copies in each sample. Numbers in red indicate limit of detection (gray symbols). Error bars indicate SEM. Shaded areas represent time with plasma HIV-1 RNA above the limit of quantification. Black arrow highlights higher viremia in P4. na, not available.

The above results indicate that proviruses with 5′-L defects precluding replication can cause NSV. To study the genesis and persistence of the T cell clones causing NSV, we performed digital PCR experiments to quantify the frequency and percentage of all infected cells belonging to *ADK*.d22 or *DNAJB14*.d21 clones (Figure 6, A and B, and Supplemental Figure 9; see Methods). We analyzed longitudinal samples collected from P1 before (3.8 years on ART) and after the onset of NSV (4.9, 6.5, 7, and 7.8 years on ART, respectively; Figure 6C). Although LTR copies remained stable (range, 463–595 copies per 10^6^ CD4^+^ T cells, *t* test between the first and last time point, *P =* 0.11), *ADK*.d22 copies significantly increased upon the onset of NSV (Figure 6C) from below the limit of detection (less than 1.13 copies per 10^6^ cells) to a new plateau of about 50 copies per 10^6^ CD4^+^ T cells. *ADK*.d22 contributed to 17% of all LTR copies — about 1 out of 3 HIV-infected cells, if we assume most proviruses have both LTRs — and with an estimated total-body clone size of 10^7^ cells (see Methods). For P2, *DNAJB14*.d21 had a frequency of 83 and 119 copies/10^6^ cells, corresponding to 2.2% and 3.3% of all LTR copies at 2 time points during NSV. This reflects the much larger pool of total infected cells compared with those from P1. Despite this small proportion, *DNAJB14*.d21 reached an estimated total body size of 24 million cells. In P3 and P4, the defective proviruses causing NSV had a frequency of 20 and 44 copies/10^6^ cells, representing only 0.5% and 1.8% of all LTR copies, respectively, and total body sizes of 4 and 6.7 million cells. Of note, infected clone sizes poorly correlated with plasma viremia. Taken together, these results show that infected clones contributing to NSV are stable and have large total body sizes, suggesting that only a small, constant fraction of these cells produce virus at any given time (15, 25).

### Proviruses causing NSV are compartmentalized in effector memory T cells.

**Figure 7:**
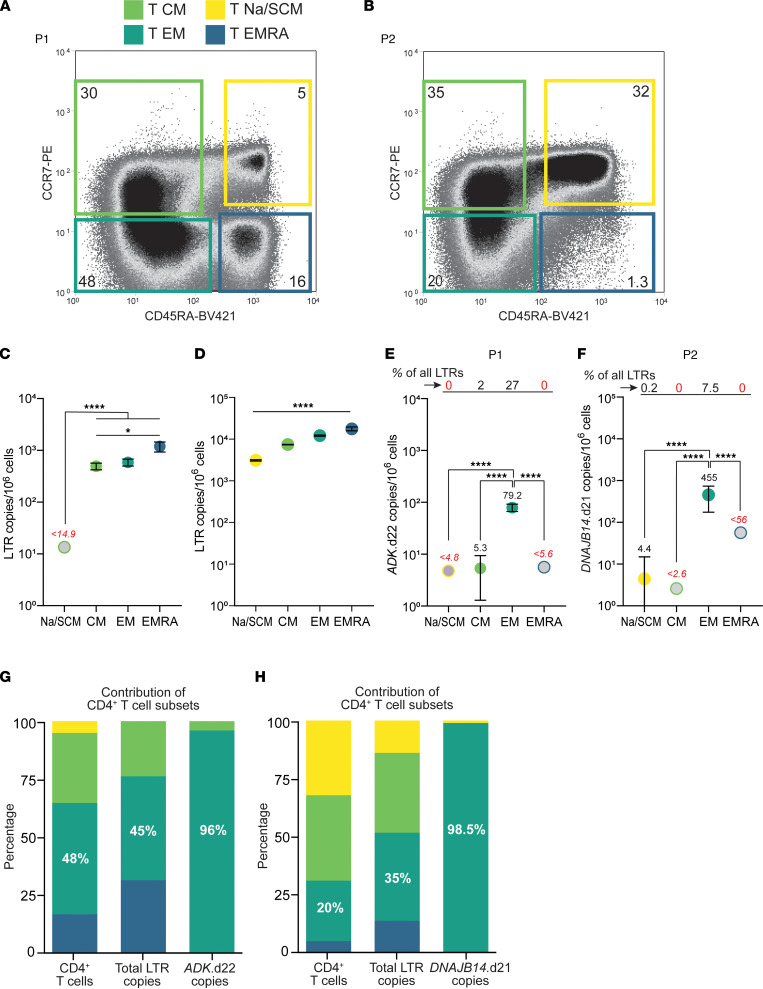
Cells carrying the *ADK*.d22 and *DNAJB14*.21 proviruses are compartmentalized in EM cells. (**A** and **B**) Distribution of CD3^+^CD4^+^ live cells based on CD45RA and CCR7 surface markers. Colored boxes represent sorting gates, and numbers indicate percentages of events in each gate. (**C** and **D**) Frequency of total LTR copies among sorted CD4 subsets. (**E** and **F**) Frequency of *ADK*.d22 and *DNAJB14*.d21 among sorted CD4 subsets. Numbers above symbols indicate mean values. Percentages of all LTRs represented by the 2 proviruses are displayed above the graph. (**G** and **H**) Contribution of subsets to CD4^+^ T cells, total LTR copies, and clones carrying the provirus of interest. Gray symbols indicate values below the limit of detection. Error bars indicate SEM. Statistical significance of differences among sorted populations was tested by 1-way ANOVA. Samples were collected at 7.8 and 26.6 years on ART, respectively. *P < 0.05; ****P <0.0001.

To investigate the distribution of proviruses causing NSV among CD4^+^ T cell subsets, we quantified LTR and integration site copies in subsets identified based on CCR7 and CD45RA expression (Figure 7). P1 showed a significant shift in T cell subset percentages relative to the expected range for individuals of his age (59) (Figure 7A), with a marked increase in the more differentiated effector memory (EM) and effector memory CD45RA^+^ (EMRA) cells, which also had a higher level of infection as assessed by LTR copies (494, 587, and 1,170 copies per 10^6^ cells in central memory [CM], EM, and EMRA cells, respectively, *P =* 0.04, Figure 7C). Interestingly, the *ADK*.d22 copies were found almost exclusively in EM cells (79.2 copies per 10^6^ cells), which contributed to 96% of the cells carrying this provirus (Figure 7, E and G).

P2 showed the expected distribution of subsets (Figure 7B) and a higher frequency of LTR copies in more differentiated cells (*P <* 0.0001) (Figure 7D). The *DNAJB14*.d21 provirus was also significantly enriched in EM cells (455 copies per 10^6^ cells, *P <* 0.0001), in which this provirus represented 7.5% of all LTR copies, while it was not found in CM and EMRA cells (Figure 7F). The EM subset contributed to more than 96% of cells carrying *DNAJB14*.d21 (Figure 7H). Thus, both clones causing NSV are compartmentalized EM cells. Given that these cells are characterized by shorter half-lives (60), their maintenance relies on frequent proliferation and differentiation from CM progenitors. Thus, it was surprising that *ADK*.d22 and *DNAJB14*.21 proviruses were not found in CM and EMRA cells, suggesting that the 2 clones may defy the canonical “differentiation flux” from central to terminally differentiated memory cells (61, 62). To investigate whether this observation is common among all CD4^+^ T cells and not unique to the 2 infected clones causing viremia, we analyzed TCR-β repertoires from total, CM, and EM cells from P1 and found that about 20% of TCR-β sequences were unique to EM cells (see Supplemental Results and Supplemental Figure 10).

We and others have shown that infected cells can persist through extensive proliferation in response to viral antigens (42, 63–65). We sorted CD4^+^ T cells reactive to CMV and HIV-1 Gag to determine whether the cells harboring *ADK*.d22 were specific to these antigens. Although P1 showed responses to both CMV and Gag (6.7% and 1.6% of all CD4^+^ T cells, respectively; Supplemental Figure 11), we did not find *ADK*.d22 within these antigen-reactive cells. Thus, the antigen specificities of the *ADK*.d22 clone and those causing viremia from P2, P3, and P4 remain unknown.

### Defective proviruses causing NSV evade cellular and humoral immune pressures.

**Figure 8:**
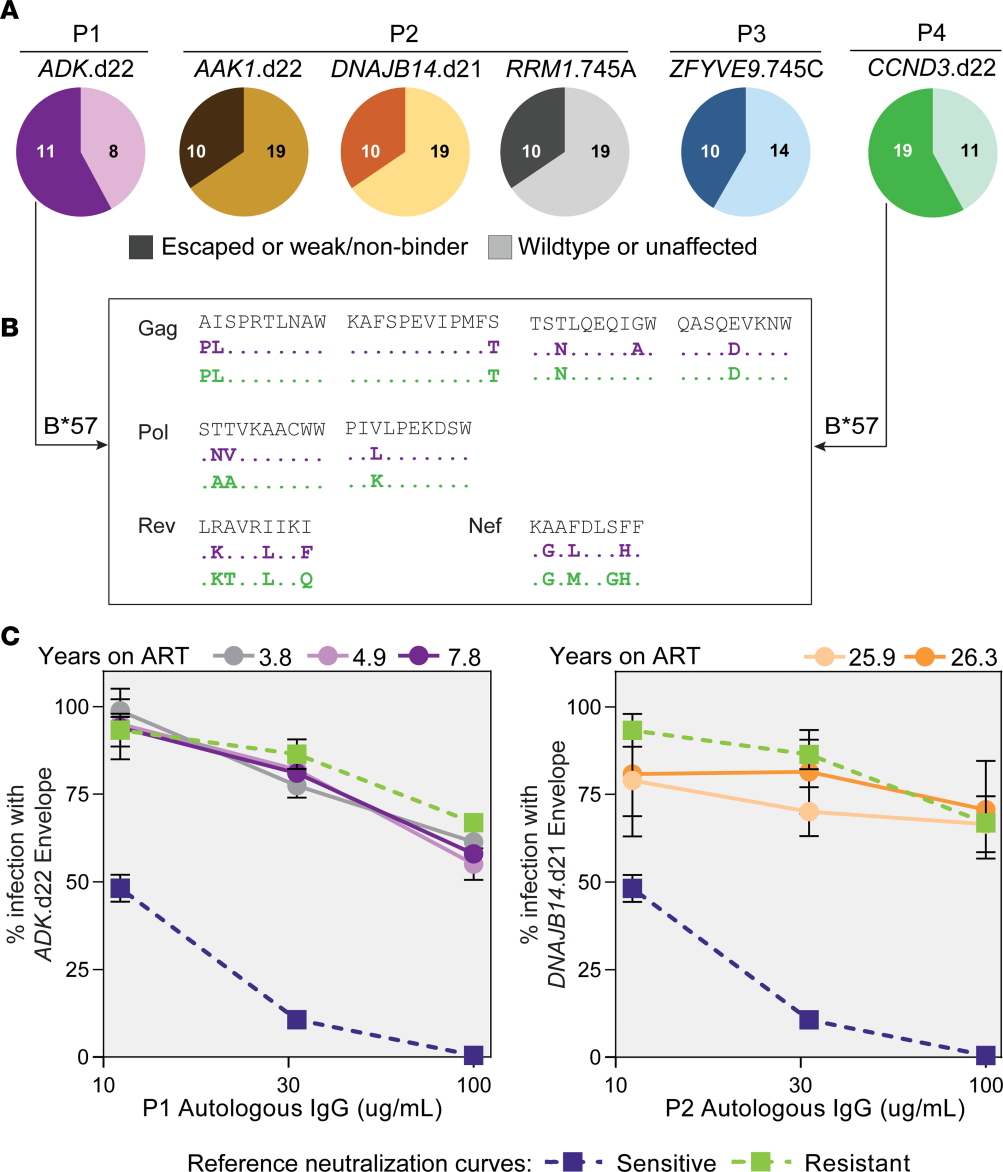
Proviruses contributing to viremia show variable proportion of CTL escape mutations and are resistant to autologous neutralization. (**A**) Distribution of T cell epitopes from full proviral genomes. Documented escape mutations and predicted weak or nonbinders are indicated in darker shades (left section of the pie charts). Numbers within pie charts represent the number of epitopes analyzed in each category. (**B**) Representative epitopes restricted by HLA-B*57 showing mutations with documented impact on HLA binding. (**C**) Neutralization experiments with autologous IgGs and viruses pseudotyped with envs from proviruses of interest. Error bars indicate SD of 3 technical replicates. Neutralization curves of stereotypical sensitive (blue) and resistant (green) envs are displayed for reference.

We hypothesized that a lack of clearance by the immune system of both virus-producing cells and virions could favor the occurrence of NSV. To investigate cytotoxic T lymphocyte (CTL) escape, we analyzed full-length genomes of proviruses contributing to NSV and identified well-characterized HLA class I restricted epitopes (Figure 8A). In P1, *ADK*.d22 had escape mutations in 8 out of 19 epitopes (58%). This was particularly evident for B*57-restricted epitopes, as 9 out of 11 (82%) had previously documented mutations that arise as a result of CTL escape (Figure 8B), including the A1P and I2L mutations in the Gag epitope AW10 and the T242N mutation in the Gag epitope TW10, the latter known to reduce viral fitness (Figure 8B and Supplemental Table 4) (66–68). The high frequency of CTL mutations is likely the result of virus evolution during more than 20 years of untreated infection before P1 started ART. A similar pattern was observed for *CCND3*.d22 from P4, in which 80% of B*57-restricted epitopes had nearly identical escape mutations (Figure 8B). Proviruses from P2 and P3 also showed some escape mutations, but retained approximately 60% of susceptible epitopes (Figure 8A).

We also evaluated susceptibility of the *ADK*.d22 and *DNAJB14*.d21 envs to autologous neutralizing antibodies (see Supplemental Results) and found that both were resistant (Figure 8C). These results suggest that persistent low-level viral expression did not elicit neutralizing antibodies over time, and future studies should investigate whether this observation is unique to defective plasma clones possibly due to low env incorporation or also typical of replication-competent proviruses causing NSV (15).

Together, these results show that defective proviruses contributing to NSV present variable degrees of CTL escape and were not neutralized by autologous IgGs. Owing to the small sample size of proviruses causing NSV and the lack of data from other intact or defective proviruses not found in plasma, we were not able to determine whether immune escape plays a fundamental role in the selection of proviruses driving NSV.

## Discussion

A small fraction of proviruses are reactivated each day, giving rise to residual viremia (6, 69) that can be detected with ultrasensitive assays in most infants and adults on ART (70, 71). In some individuals, this process results in years of detectable viremia produced by expanded clones carrying infectious proviruses (14, 15). The true frequency of NSV during ART is unknown. However, based on the study from Halvas and colleagues (15), and the cases referred from our clinic, we estimate that 1 in approximately 250 individuals on ART experiences persistent NSV. These cases are extreme examples of how the reservoir persists in all individuals on ART (16, 18, 19), but which factors lead a handful of infected clones — out of a myriad — to cause NSV remain unknown.

Some defective proviruses can produce HIV-1 RNA and viral proteins (26, 28, 29, 72), potentially leading to inflammation and/or immune activation and deflecting CTL responses (26, 29, 30). However, whether defective proviruses can cause NSV has been unknown. A previous report described defective viral variants in the context of residual viremia below 50 copies/mL (73). Here, we describe 4 consecutive cases of PLWH on long-term ART with persistent NSV of up to 10^3^ copies/mL due to 1 or multiple proviral clones with defects in the 5′-L. In participants P1, P3, and P4, plasma virus was entirely caused by a single defective provirus, while P2, who had a large reservoir, a mixture of infectious and 5′-L–defective proviruses contributed to NSV. All 6 defective genomes described here had alterations affecting the MSD site. Surprisingly, proviruses from 3 distinct participants shared the same d22 deletion, likely favored by the “GAG” repeats driving misplaced recombination events during reverse transcription (47). The remaining 2 proviruses had single nt mutations at the critical T in the MSD (D1) site: T745A in P2, and T745C in P3, both previously reported (33, 74) and associated with a loss of replication competence (73). Both mutations affect the second position of the conserved “GU” dinucleotide immediately downstream of the splicing site. The mutated nt is included in the GURAGU motif recognized by the U1 snRNA, which is required to initiate the splicing process (75). Of note, besides these 5′-L mutations, these 6 proviruses described here had genetically intact promoters and open reading frames, including the antisense protein ASP, which has been shown to promote latency (76).

Although nearly all 5′-L defects abrogate replication competence, their variable size and position complicate prediction of which 5′-L functions are disrupted. Das et al. proposed that MSD-Ѱ mutations could prevent proviral expression by activating the 5′-polyadenylation site, resulting in ultrashort noncoding HIV-1 transcripts (77). However, previous transfection experiments (26), assays based on T cell stimulation ex vivo (33), and the data presented here demonstrate that proviruses with 5′-L deletions can be induced and can produce virions. Our studies of dimerization and capsid binding show that these functions are only partially affected by the 5′-L mutants causing NSV. Experiments with competition-based packaging assays and incorporation of radio-labeled dNTPs may tease out more granular differences in efficiency relative to intact proviruses (78). However, our results show that these proviruses can contribute to viremia at levels that can be detected by clinical assays despite modest dimerization and packaging defects.

To maintain replicative fitness, HIV-1 must exploit the full spectrum of its alternative splicing possibilities (50). Deviations from the delicate balance between unspliced and spliced mRNAs can influence virus replication (79). The MSD site (or D1) plays a fundamental role in the regulation of HIV-1 splicing and is found in nearly all spliced transcripts (50). However, alternative splicing donors have been described (51) and can take over if D1 is mutated or missing (26). We confirmed this phenomenon by sequencing spliced mRNA forms in CD4^+^ T cells from P1, in which the 5′-L deletion found in *ADK*.d22 created a donor found in more than 90% of spliced transcripts. However, this alternative donor did not fully rescue generation of all HIV-1 mRNAs. We did not detect spliced mRNAs coding for Rev, which is fundamental for efficient nuclear export of intron-retaining viral transcripts (50). Most importantly, although we detected spliced RNAs encoding Env in P1 cells stimulated ex vivo, the d22 and d21 deletions caused a striking reduction in Env expression in transfected cells that mirrored the lack of gp160/gp41 incorporation in viral particles.

Longitudinal studies of PLWH on long-term ART have shown that proviral populations are dynamic and under ongoing selective pressures, resulting in the more rapid decay of intact proviruses relative to proviruses with major defects (24, 80–83). The high frequency of 5′-L defective proviruses in individuals on ART, both within the proviral landscape overall and among highly expanded clones, suggests that these nearly intact proviruses have a selective advantage relative to intact proviruses, even if frequently expressed. 5′-L defects such as d22 and d21 may allow the production of viral particles upon latency reversal while maintaining lower levels of viral proteins involved in cytopathic effects and immune recognition, especially env. We hypothesize that 5′-L defective proviruses may be a common cause of residual viremia. In the 4 participants described here — who were not preselected — 1 or more 5′-L defective proviruses contributed to NSV. The development of clinical ultrasensitive assays that sequence the 5′‑L as well as the *pol* gene would improve care of individuals with NSV by reducing concerns over cryptic viral replication and risk of HIV-1 transmission, unnecessary laboratory tests, and changes in ART regimens (84).

The genomic context of HIV-1 integration plays a role in the inducibility, proliferative potential, and persistence of infected cells (28, 85). The 6 integrants causing NSV described here were all located in gene-rich regions, within introns of coding genes in opposite orientation relative to their host gene transcription. Five out of six were within 20 kb of known H3K27Ac and H3K4Me1 and H3K4Me3 histone marks, associated with enhancer regions in uninfected cells (86), and in genes expressed at medium-to-high levels in CD4^+^ T cells, involved in housekeeping functions such as nt metabolism, DNA damage response, vesicle-mediated transport, and cell-cycle progression. The exception was the *ZFYVE9.*745C provirus from P3, which is integrated into a gene expressed at very low levels in CD4^+^ T cells. Overall, although HIV-1 integration in these genes has not been linked to a selective advantage, these proviruses are likely located in genomic loci favorable for HIV-1 gene expression (28).

Clonal proliferation is a major mechanism of persistence of cells carrying both intact and defective proviruses (85). Here, we directly quantified 4 clonal proviruses by integration-specific digital PCR assays. Total body clone sizes — on the order of 10^6^ to 10^7^ cells — were comparable to those observed in HIV-1^+^ CMV-responding clonotypes and clones contributing to NSV (15, 25, 42). Interestingly, *ADK*.d22 represented about 30% of all infected cells and 50% of all *env* proviral sequences in P1. This finding is in striking contrast with previous studies, in which proviruses matching the predominant plasma clones are rarely found in resting CD4^+^ T cells (10), and represented a small fraction of HIV-1 DNA single-genome sequences (0%–10.7%) and integration sites (0.03%–1.10%) (15). The high proportion of *ADK*.d22 proviruses can be explained by the small pool of infected cells in P1, a B*57^+^ viremic controller who remained off of ART for more than 20 years. This observation is in line with reservoir studies from elite controllers in which few large clones dominate the proviral landscape (87, 88). For what we believe is the first time, we show that the onset of viremia is concurrent with the rapid waxing of the cells containing the *ADK*.d22 provirus, suggesting an immune event that activated its cognate clonotype, leading to proliferation and frequent virus production. In addition, our results on *ADK*.d22 and *DNAJB14*.d21 indicate that they are stable and compartmentalized in short-lived EM cells, suggesting persistence by frequent proliferation, rather than homeostatic survival signals and long half-lives (60). Immune responses to recurrent antigens, such as HIV-1 and cytomegalovirus, can drive the proliferation of infected clones (42). However, the cognate antigens of the clones causing NSV are yet to be determined. NSV may be a reflection of specific T cell responses against antigens that cannot be cleared, such as chronic infections, including HIV-1 itself, commensal pathogens, or self-antigens. This may explain why only rare proviruses are frequently transcriptionally active, including those that will ultimately lead to viral rebound upon treatment interruption (33, 89, 90).

Taken together, our results are consistent with the finding that NSV is due to 5′-L defective viruses produced from the same provirus-containing clones; however, given the lack of sampling at multiple time points before and during ART documenting the same integration sites, alternatives cannot be excluded. These include the production of virus with limited diversity from different cells or rare new infection events with reseeding of new virus-producing cells over time. In addition, future studies should include a systematic comparison between defective and infectious proviruses causing NSV in order to tease out unique features, including susceptibility to cell-mediated and humoral immunity, resistance to cell death, and genomic location.

In conclusion, we demonstrate that proviruses with 5′-L defects, when in favorable genomic and immunological conditions, are a frequent cause of persistent NSV despite effective ART. Although incapable of causing viral rebound, these proviruses contribute to variable levels of viremia and complicate ART management (84), leading to additional testing, unnecessary ART changes and intensifications, and causing anxiety and frustration in patients and clinical care providers. Our work reveals additional complexity in residual viremia and should prompt the development of assays that would allow detection of 5′-L defects.

## Methods

### Study participants.

The study participants were referred to us by their HIV-1 care providers at the Bartlett Specialty Clinic, Johns Hopkins University (P1–P3), and at the Clinique I.D. of Saint-Jérôme (Quebec, Canada). Peripheral blood samples (180 mL) were collected at 1 or multiple time points (2019–2022). For P1, historical samples (2013–2018) were obtained through a longitudinal study at the Bartlett Specialty Clinic. Samples from P4 were collected at CHUM.

### Study of HIV-1 sequences in plasma and CD4^+^ T cells.

Blood samples were spun at 400*g* for 10 minutes at 4°C and plasma was spun again at 400*g* for 10 minutes and frozen at –80°C. Upon thawing, plasma was spun at 3,500*g* for 15 minutes at 4°C, transferred to tubes for ultracentrifugation, and spun at 170,000*g* for 30 minutes at 4°C. Viral pellets underwent RNA extraction (91). RNA was used immediately for reverse transcription with SuperScript III with primers located in *gag*, RT, or *env*. The cDNA was then used for single genome sequencing as previously described (42). For all participants, we initially recovered p6-RT sequences to exclude drug resistance. For P1, due to the small reservoir size and the partial control of HIV replication before ART, we focused on env SGS to avoid the risk of overestimating clonal sequences.

### IPDA.

IPDA was performed as previously described (24).

### QVOA.

QVOAs from total CD4^+^ T cells were performed as previously described (46). Supernatants from p24-positive wells were processed as previously described to sequence virion-associated HIV-1 RNA (42).

### Combined analysis of integration site and proviral genome.

Endpoint-diluted gDNA was subjected to whole-genome amplification, as previously described (92). Wells positive for HIV-1 genomes were detected by *gag*, *env*, or p6RT PCR. Wells with defective proviruses matching the predominant plasma clones were subjected to integration site analysis and near full-length genome sequencing (92). Additional PCRs were performed to confirm the site of HIV-1 integration and recover the LTR sequence. Primers are provided in Supplemental Table 5.

### Analyses of HIV-1 sequences.

Sanger sequencing data were processed and analyzed as previously described (42). In brief, neighbor joining trees were performed based on a *p* distance and bootstrap analysis with 1,000 replicates. For the analysis of pre-ART sequences from P1 and sequences from P2 shown in Figure 1, we used maximum likelihood method based on the HKY+G substitution model with 1,000 replicates, identified with Mega, version 7.0. For the tree analysis on near full genomes in Supplemental Figure 2D, we used a GTR+G+I substitution model. Intactness of proviral full-length genomes was assessed by Proseq-IT (93).

### 5′-L RNA studies.

Methods can be found in Supplemental Methods.

### Testing the impact of 5′-L deletions.

We used site-directed mutagenesis (New England Biolabs) to introduce each 5′-L deletion into an NL4-3 backbone, obtained through the NIH HIV Reagent Program, Division of AIDS, NIAID: ARP-114, contributed by M. Martin. Plasmids carrying the WT (NL4-3wt) and mutants (NL4-3d22 and NL4-3d21) were used to transfect 293T cells (26). After 72 hours, we collected the supernatant, which was then filtered and concentrated by ultracentrifugation with a 20% sucrose gradient. Virus recovery was measured by p24 ELISA (PerkinElmer) and reverse transcriptase PCR (RT-PCR) measuring polyadenylated HIV-1 RNA. We then infected primary CD4^+^ T cells from a healthy donor after 3 days of activation with anti-CD3/anti-CD28 antibody–coated beads (1:1 cell to bead ratio). Each condition was tested in triplicate by spinoculation, with 10 ng of p24 per 1 million cells in 100 μl of media. After spinoculation, cells were washed 5 times with ice-cold PBS and plated at 1M/mL in RPMI with 10% FBS and 20 U/mL f IL-2. To control for background p24 and plasmid carryover, an identical experiment was carried out in parallel with media containing tenofovir disoproxil fumarate (10 μM), emtricitabine (10 μM), and dolutegravir (10 nM). Culture supernatants were collected at 0, 12, 24, 48, and 72 hours after spinoculation and assayed by p24 ELISA. Cells were collected at 0, 6, and 12 hours after spinoculation to extract genomic DNA and quantify late reverse-transcriptase cDNA products by droplet digital PCR (ddPCR) probing the U5-PBS junction. Cell-associated RNA at 24 hours after 293T cell transfection underwent cDNA synthesis as described above and was quantified by digital PCR (QIAGEN) targeting all polyadenylated RNA and spliced mRNA belonging to the 4 kb class and encoding for tat/rev (Supplemental Table 5). To capture mRNA from both WT and mutant viruses, the 4 kb class assay was designed to capture mRNA regardless of the D1 splicing donor site.

### Western blots.

Information can be found in Supplemental Methods.

### Flow cytometry analysis of cells expressing HIV-1 env.

Transfected 293T cells were briefly dissociated using 100 μL of TrypLE (Thermo Fisher Scientific) for 2 minutes followed by 1 wash in DMEM supplemented with 10% FBS. Cells were resuspended in 10% DMEM at a concentration of 2M/mL to seed 100 μL into 96-well V-bottom plates. Cells were incubated with unlabeled primary antibody 3BNC117 (NIAID, ARP-12474) at a final concentration of 15 μg/mL for 1 hour at 37°C. After 2 washes, cells were then stained with BV421-labeled secondary antibody against hu-IgG Fc (1:40 diluted, BioLegend, clone M1310G05) and viability dye (1:1000 diluted, eBioscience, eFluor 780) for 30 minutes at 4°C. After 2 washes to remove excess antibodies, cells were analyzed using an Intellicyt iQue cytometer. Nonspecific signal was assessed by staining cells with only BV421-labeled secondary antibody and by staining cells transfected with a delta-*env* NL4-3 expression vector (NIAID, HRP-11100). Finally, background measured by using an anti-human IgG primary antibody was subtracted from cells stained with 3BNC117.

### Transmission electron microscopy.

Information can be found in Supplemental Methods.

### Analysis of HIV-1 expression upon T cell activation.

Total CD4^+^ T cells were isolated from PBMCs by negative selection and plated in 24-well plates at 2 million/mL in RPMI with 10% FBS, 10 nM dolutegravir, and anti-CD3/CD28 antibody–coated magnetic beads (cell-to-bead ratio, 1:1). Cells and culture supernatant were collected at 0, 24, and 48 hours. Cell-associated genomic DNA and RNA were extracted as previously described (94). RNA fractions were subjected to cDNA synthesis as described above using a gag-specific primer. We used 3 probe-based assays to quantify the following: (a) total HIV RNA (RU5) from any provirus, (b) unspliced RNA from defective proviruses of interest using probes annealing across the 5′-L deletion (Supplemental Figure 6); and (c) read-through RNA from upstream of the LTR using probes annealing across the provirus-specific integration site. For P1, we generated cDNA from cell-associated RNA in 2 separate reactions using primers annealing downstream of splicing acceptors A5 and A7 to capture 2 kb and 4 kb transcripts, respectively (52). Subsequently, cDNA was subjected to limiting-dilution 2-step PCR to amplify singly and multiply spliced transcript using forward primers upstream of both canonical and alternative splicing donors. See Supplemental Table 5 for additional details.

### Duplex quantification of total LTR copies and specific provirus.

Information can be found in Supplemental Methods.

### T cell subset analysis.

Information can be found in Supplemental Methods.

### Analysis of HLA-restricted epitopes.

Participants were HLA typed at high resolution at the Johns Hopkins University Immunogenetics Laboratory. The CTL epitopes for each participant were identified using the Los Alamos National Lab’s Best-defined CTL/CD8^+^ Epitope Summary (https://www.hiv.lanl.gov/content/immunology/variants/ctl_variant.html). When functional annotations of certain mutations were not available, we used the prediction software NetMHC4.0 to classify epitopes as strong, weak, or nonbinder (95).

### Neutralization assay.

The complete sequence of the *env* gene was recovered as previously described (96). The starting material was viral RNA isolated from supernatant of autologous CD4^+^ T cells activated ex vivo for 24 hours with anti-CD3/CD28 beads (as described above). The PCR products, matching 100% of the *ADK*.d22 and *DNAJB14*.d21 proviruses, were cloned into an *env* expression plasmid and used to cotransfect HEK293T cells together with an *env* deleted NL4-3 vector, to generate pseudotyped virus. Pseudoviruses were titrated and assayed for neutralization in TZM-bl cells as previously described (96). Inhibition of infection was expressed as a fraction of maximum infection. IC_50_ was calculated as previously described (97).

### Isolation of antigen-reactive CD4^+^ T cells.

Experiments were conducted as described in Simonetti et al. (42).

### Data availability.

HIV-1 sequences are available in the NCBI’s GenBank database (OQ092462, OQ092467, OQ458809, OQ458982, OQ458983, OQ459106, OQ459107, OQ459244, OQ459245, OQ459280). Integration site sequencing data can be found on the NCI Retrovirus Integration Database (https://rid.ncifcrf.gov/; Supplemental Table 2). TCR-β sequencing data can be accessed through the ImmuneAccess database (https://doi.org/10.21417/JAW2023JCI).

### Statistics.

Descriptive statistics, tests for normality, 2-tailed Student’s *t* test, and 1-way ANOVA were used to determine statistical significance using GraphPad Prism, version 8.0. A *P* value of less than 0.05 was considered significant, unless otherwise stated.

### Study approval.

The Johns Hopkins Institutional Review Board approved this study. Study participants provided written, informed consent before enrollment. For P1, historical samples (2013–2018) were obtained through an Institutional Review Board–approved longitudinal study at the Bartlett Clinic. P4 provided written consent for an Institutional Review Board–approved study from the laboratory of Cécile Tremblay at CHUM.

## Author contributions

FRS, JDS, and RFS conceived the study. FRS, JAW, FW, MM, JD, FD, ACC, and KR performed experiments and analyzed data. SAB and JL enrolled study participants and performed sample processing and viral outgrowth assays. SY, MYZ, NFT, HBP, JMCQ, and MFS conceived and performed the study of 5′-L RNA structure and function. CJH, CK, JC, NC, PBC, RM, SP, FC, and CT provided clinical care to the study participants and gathered their clinical history and samples. HZ conducted cell-sorting experiments. JV performed pseudotyped virus neutralization assays. MD prepared samples and acquired electron microscopy images. FRS conducted analyses and generated figures. FRS wrote the manuscript with input from JDS and RFS and received feedback and final approval from all authors. JAW and FW contributed equally to this work, and their order as co–first authors is alphabetical.

## Supplementary Material

Supplemental data

Supplemental uncut gels

ICMJE disclosure forms

## Version 1 01/05/2023

In-Press Preview

## Version 2 03/15/2023

Electronic publication
