# Supplementary material for: Clonally expanded HIV-1 proviruses with 5′-leader defects can give rise to nonsuppressible residual viremia
Source: J Clin Invest. 2023 Mar 15;133(6):e165245. doi: 10.1172/JCI165245 (PMC10014112; doi:10.1172/JCI165245)
Supplement: Supplemental data [file jci-133-165245-s076.pdf]

Clonally expanded HIV-1 proviruses with 5'-Leader defects can give rise to nonsuppressible residual viremia.

Jennifer A. White*, Fengting Wu*, Saif Yasin, Milica Moskovljevic, Joseph Varriale, Filippo Dragoni, Angelica Camilo Contreras, Jiayi Duan, Mei Y. Zheng, Ndeh F. Tadzong, Heer B. Patel, Jeanelle Mae C. Quiambao, Kyle Rhodehouse, Hao Zhang, Jun Lai, Subul A. Beg, Michael Delannoy, Christin Kilcrease, Christopher J. Hoffmann, Sébastien Poulin, Frédéric Chano, Cécile Tremblay, Jerald Cherian, Patricia Barditch-Crovo, Natasha Chida, Richard Moore, Michael F. Summers, Robert F. Siliciano, Janet D. Siliciano, Francesco R. Simonetti.

Supplementary Materials

<u>**Table of contents**</u>

Supplementary Results
-Extended clinical history of study participants.
-Integration site analysis of proviruses causing viremia.
-CD4+ T cell repertoire analyses from participant P1.
-Analysis of autologous neutralization

Supplementary Methods
-5'-Leader RNA studies.
-Western blots.
-Transmission Electron Microscopy.
-Duplex quantification of total LTR copies and specific provirus.
-T cell subset analysis.

Supplementary References

Supplementary Figures
Figure S1. Clinical history and HIV-1 population analysis of Participant 3.
Figure S2. Clinical history and HIV-1 population analysis of Participant 4.
Figure S3. Additional HIV-1 sequence analyses related to Figure 1.
Figure S4. Viral outgrowth assay from P1 shows lack of exponential outgrowth.
Figure S5. 5'-Leader deletions cause a modest decrease in the number of nucleocapsid binding sites.
Figure S6. Validation of digital PCR assays that selectively amplify proviruses of interest, related to Figure 4.
Figure S7. Deletions do not alter tRNA binding propensity to the 5'-Leader.
Figure S8. Production of viral particles upon transfection with 5'-Leader-defective NL4-3.
Figure S9. Impact of controlled shearing on genomic DNA used in integration site-specific digital PCR assays.
Figure S10. TCR analyses of CD4+ T cell central and effector memory subsets.
Figure S11. Isolation of antigen-responding CD4+ T cells from PBMCs in P1.

Supplementary Tables
-Table S1. Study participant characteristics.
-Table S2. Integration site analysis of proviruses contributing to NSV.
-Table S3. ITC isotherm fitting data of NC to NL4-3 WT, d21, d22 to full saturation.
-Table S4. Analysis of CTL mutations.
-Table S5. Oligos used in this study.

Supplementary Results

Extended clinical history of study participants. P1 is an HLA-B*57:03$^+$ slow-progressor who initiated ART with a CD4 nadir of 454 cells/µL more than 20 years after HIV-1 diagnosis. Over time, he developed comorbidities including type-2 diabetes, hypertension, mixed hyperlipidemia, and hypogonadism. At 6.8 years on ART, he had an HIV-1 reservoir size significantly below average (<0.06 infectious units per million (IUPM) by the quantitative viral outgrowth assay (QVOA) (1), and 3.5 copies of intact proviruses/10^6 total CD4+ T cells by the intact proviral DNA assay (IPDA) (2). P2 had a diagnosis of HIV-1/AIDS in 1991, a nadir of 197 cells/µL, and has been on ART for more than 26 years. He had a Roux-en-Y gastric bypass in 2010 and a history of hyperlipidemia, hypogonadism, and benign prostatic hyperplasia. His reservoir size falls on the opposite side of the spectrum, with 15 IUPM by QVOA and 311 intact proviruses/10^6 total CD4$^+$ T cells. P3 initiated ART shortly after diagnosis in 2007 with a nadir of 221 cells/µL and maintained an undetectable viral load for almost 10 years. Her reservoir size falls within the average of most individuals who started ART during chronic infection (161 copies of intact proviruses/10^6 total CD4$^+$ T cells) (3). P3 is on chronic treatment with azathioprine due to type-I autoimmune hepatitis. P4 had a diagnosis of HIV-1/AIDS and Kaposi's Sarcoma around 1990 and has been on ART for more than 26 years. Although medical records before 2016 are limited, he maintained prolonged suppression until 2020, when his viral load reached a plateau on the order of 10^3 copies/mL. This was initially interpreted as virological failure, and his clinical care providers thoroughly assessed drug resistance, drug concentrations, and adherence, which were all unremarkable. ART optimization, intensifications, and one month of directly observed therapy failed to decrease NSV. His comorbidities include type-2 diabetes, psoriasis, and bilateral nephrolithiasis. P4's reservoir size is also within the typical range of PLWH on ART (58 intact proviruses/10^6 total CD4$^+$ T cells) (3). At the time of this report, all four participants are in overall good health and show stable CD4$^+$ T cell counts above 600 cells/µL, while HIV-1 RNA in plasma remains above 20 copies/mL despite ART.

Integration site analysis of proviruses causing viremia. The site of HIV-1 integration into particular host genes, for example host genes involved in cell proliferation, may affect the persistence of infected cells as it can lead to aberrant expression of the host gene in a way that promotes the survival and proliferation of a clone of infected cells (4). In rare cases, the site of HIV-1 integration can contribute to silencing of the integrated provirus (5). In addition, the analysis of HIV-1 sub-genomic sequences can lead to an overestimation of proviral clonality (6, 7). Therefore, we s confirmed the clonal nature of proviruses causing viremia by integration site analysis. Through paired provirus and integration site data based on limiting dilution and whole genome amplification (7, 8), we characterized proviruses matching variants found in plasma (Figure 2C and Supplementary Table S2) from total or effector memory cells, as the latter have been linked to higher infection frequency and clonal expansion (9-11). The predominant plasma clone found in P1 is integrated the first intron of the Adenosine Kinase (*ADK*) gene, downstream of the translation start site of both long and short *ADK* splice variants (nuclear and cytoplasmic forms, respectively). In P2, we recovered the integration sites for 3 proviruses causing viremia at one or more time points. These proviruses were located in the *AAK1*, *DNAJB14*, and *RRM1* genes (see Supplementary Table, S1). In uninfected individuals, these four genes show inducible expression at medium to high levels in CD4$^+$ T cells. The provirus causing NSV in P3 is integrated within the *ZFYVE9* gene, which encodes for a zinc finger-containing protein involved in TGFβ signaling, is poorly expressed in CD4$^+$ T cells. In P4, the provirus responsible for NSV is integrated into the *CCND3* gene, a highly expressed gene involved in G1/S phase transition and cell proliferation. Interestingly, all 6 proviruses were integrated in opposite orientation relative to the host gene transcription, a proviral feature that is selected for in

individuals on long-term ART (12, 13). Viral integration in these genes have been previously identified both in infection *in vitro* as well as in PLWH before and on ART. However, none of these genes have been linked to HIV-1 persistence due to insertional mutagenesis (12, 14), suggesting that immune stimuli are the main drivers of their proliferation and viral production.

CD4⁺ T cell repertoire analyses from Participant 1. To investigate whether compartmentalization within effector memory cells is common among all CD4⁺ T cells, and not unique to the two infected clones causing viremia in P1 and P2, we analyzed TCRβ repertoires from total, CM, and EM cells from P1 (~6000 productive TCRβ sequences per sample). While CM cells showed striking richness, EM cells had the highest degree of clonality, as previously described (15). This latter subset was dominated by very large clones: the top 50 clonotypes ranked by abundance contributed to more than 60% of all sequences, and the most expanded clone among all CD4⁺ T cells (CASSDLGQGHTEAFF, 11%) represented 24% of all CDR3β sequences in EM cells (~1 cell out of 4), compared to only 0.2% in the CM subset Supplementary Fig S9B). We then compared TCRβ sequences found in both CM and EM subsets versus those found solely in EM cells (4027 versus 9010 unique sequences, respectively, supplementary Figure S9D). Sequences found in both subsets were on average significantly more abundant in EM cells, supporting the hypothesis of a differentiation-proliferation flux directed from CM to EM (16) (Figure S9E). TCRβ sequences found only in the EM subset had a cumulative abundance of 21% among all EM cells, but were significantly less expanded than total EM clonotypes ($p<0.0001$, Figure S9 F and G), likely a reflection of the lower proliferative capacity of EM clonotypes not relying on differentiation from CM cells.

Analysis of autologous neutralization. Antibody-mediated immune pressure can potentially affect HIV-1 reservoir dynamics, including the selection of which proviruses lead to viral rebound upon treatment interruption (17). Our site-directed mutagenesis experiments suggest that the small 5'-L deletions in *ADK*.d22 and *DNAJB14*.d21 proviruses resulted in reduced Envelope levels in virus-producing cells and virions (Figure 5E-I). However, low-level Env expression on productively-infected cells could still result in engagement by neutralizing and/or effector antibodies and affect cell survival (18, 19). Therefore, we investigated whether viruses pseudotyped with *ADK*.d22 and *DNAJB14*.d21 full-length Envelopes could be neutralized with autologous IgG in a TZM-bl cell-based assay, as previously described (17). As shown in Figure 8C, the Envelope from both proviruses is infectious but substantially resistant to autologous neutralization. At the latest time points, inhibition of *ADK*.d22-Env and *DNAJB14*.d21-Env viral entry did not reach 50% at 100 ug/mL purified IgGs, the highest concentration tested in the assay. Plasma samples from P1 obtained before, or years after, the onset of NSV (3.8 versus 4.9, and 7.8 years on ART, respectively) showed no change in *ADK*.d22-Env neutralization (IC50 >100 ug/mL). Similarly, the lack of *DNAJB14*.d21-Env neutralization was comparable between two plasma samples collected 3 months apart (IC50 >100 ug/mL).

Supplementary Methods

Preparation of DNA Templates. The NL4-3 5′-Leader wildtype DNA template was generated from a pUC19 plasmid containing the Top17 sequence (5′-TAATACGACTCACTATA-3′) and the RNA encoding sequence (5′-
GGTCTCTCTGGTTAGACCAGATCTGAGCCTGGGAGCTCTCTGGCTAACTAGGGAACCCAC
TGCTTAAGCCTCAATAAAGCTTGCCTTGAGTGCTCAAAGTAGTGTGTGCCCGTCTGTTGTG
TGACTCTGGTAACTAGAGATCCCTCAGACCCTTTTAGTCAGTGTGGAAAATCTCTAGCAGT
GGCGCCCGAACAGGGACTTGAAAGCGAAAGTAAAGCCAGAGGAGATCTCTCGACGCAGG
ACTCGGCTTGCTGAAGCGCGCACGGCAAGAGGCGAGGGGCGGCGACTGGTGAGTACGC

CAAAAATTTTGACTAGCGGAGGCTAGAAGGAGAGAGATGGGTGCGAGAGCGTCGGTA-3′). d21 and d22 patient derived 5′-Leaders were generated by site directed mutagenesis (Q5® Site Directed Mutagenesis Kit, New England Biolabs) using forward primers: 5′-TTTGACTAGCGGAGGCTAGAAGGAGAGAG-3′ and 5′-TACGCCAAAAATTTTGACTAGCGGAG-3′, and reverse primers: 5′-CGCCGCCCCTCGCCTCTT-3′ and 5′-CTCTTGCCGTGCGCGCTT-3′, respectively. $tRNA^{Lys3}$ template was generated from a pUC57 plasmid (Genewiz) containing Top17 sequence and the RNA encoding sequence (5′-GCCCGGCTAGCTCAGTCGGTAGAGCATCAGACTTTTAATCTGAGGGTCCAGGGTTCAAGTCCCTGTTCGGGCGCCA-3′). DNA templates were generated by standard PCR amplification (EconoTaq PLUS 2x Master Mix, Lucigen) of plasmids described above. A forward amplification primer 80 nucleotides upstream of the T7 promoter were used for all constructs (5′-GGGATGTGCTGCAAGGCGATTAAGTTGGG-3′). The reverse amplification primer for WT, d21, d22 5′-Leader constructs was 5′-mUmACCGACGCTCTCGCACCCATC-3′, while constructs truncated within the AUG hairpin for ITC experiments used 5′-mCmGCACCCATCTCTCTCCTTCTAGCCT-3′ (20, 21). Truncations were used for ITC experiments to highlight the endothermic contribution associated with initial NC binding sites. Reverse amplification primer for $tRNA^{Lys3}$ was 5′-mUmGGCGCCCGAACAGGGAC-3′. Methylated reverse primers were used to reduce self-templated run on during in vitro transcription (20). All DNA templates were subsequently validated by Sanger sequencing (Eurofins Genomics).

RNA in Vitro Transcription. RNAs were prepared via T7 RNA polymerase (purified in-house) in 15-mL reactions. A 15-mL reaction contained ∼1 mg of PCR-amplified DNA template, 20 mM $MgCl_2$, 3 mM NTPs, 2 mM spermidine, 2 mM DTT, 20% (vol/vol) DMSO, 80 mM Tris·HCl (pH 9.0), and T7 RNA polymerase. Amounts of each component were optimized via small-scale (30 µL) transcription reactions. The reaction was quenched after 8-hours of incubation at 37 °C by addition of an EDTA mixture (250 mM EDTA, pH 8.0) and was boiled for 5 min, then snap cooled on ice for 5 min prior to addition of glycerol (final concentration, 6% [vol/vol]). RNAs were purified by electrophoresis on 7.5 M urea-containing polyacrylamide denaturing gels (SequaGel; National Diagnostics) at 30 W for 12 hours, visualized by UV shadowing, and eluted using the Elutrap electroelution system (Whatman) at 130 V overnight. The eluted RNAs were concentrated and washed twice with 2 M high-purity NaCl followed by extensive desalting (~40 mL Water) using Amicon Ultra Centrifugal Filter Device (Millipore).

NC Purification. $HIV\text{-}1_{NL4\text{-}3}$ NC (55 amino acids: MQKGNFRNQRKTVKCFNCGKEGHIAKNCRA PRKKGCWKCGKEGHQMKDCT ERQAN) was placed into a pET-3a plasmid and transformed into BL21 (DE3) pLysE. The protein was overexpressed and purified as previously described (22). Cells were lysed via freeze-thawing and microfluidization (6 times). Lysate was treated with 10% polyethylenimine (added 4% of lysate volume) to remove excess nucleic acids. After centrifugation, supernatant was applied to ion exchange chromatography using tandem Q- and SP- columns (Q-column was removed before elution). Final protein was isolated using size exclusion chromatography on a Superdex 30 column in 20 mM Tris·HCl, pH 7.5, 140 mM KCl, 10 mM NaCl, 5 mM $MgCl_2$, and 5 mM TCEP.

Isothermal Titration Calorimetry. ITC experiments were carried out using a MicroCal PEAQ-ITC Automated (Malvern Panalytical). To reduce non-specific NC binding and NC-induced unwinding caused by the chaperone activity of NC, titrations were conducted in the presence of 5 mM $MgCl_2$ (23, 24). A volume of 40 µL of NC (200-250 µM) in ITC buffer (20 mM Tris·HCl, pH 7.5, 140 mM KCl, 10 mM NaCl, 5 mM $MgCl_2$, and 5 mM TCEP) was loaded into the injection syringe. The calorimetry cell was loaded with 200 µL of RNA (1 µM for titration to saturation and 3 µM for assessment of initial binding) in the same buffer as NC. After thermal equilibration at 25°C and the initial 60-s delay, a single injection of 0.4 µL followed by 18 serial injections of 2

μL were made into the calorimetry cell with a 120-s delay between injections. Protein to Buffer and Buffer to Buffer controls were subtracted from data. Data was baseline corrected. The raw data integration, and curve fitting was done using Malvern's MicroCal PEAQ ITC analysis software to get stoichiometry, affinities, and thermodynamic parameters.

***In Vitro* Dimerization Assay.** NL4-3 WT, d21, and d22 5′-Leaders (12.5 μM in water) were heat denatured for 5 minutes and cooled on ice for 5 minutes to prevent nonnative higher order structures. Samples were then prepared in physiological ion buffer with 20 mM Tris·HCl (pH 7.5), 140 mM KCl, 10 mM NaCl, and 1 mM $MgCl_2$ at 10 μM RNA concentration. Samples were subsequently diluted to desired concentrations (0.1, 0.5, 1, 5 μM). 350-ng RNA samples were loaded onto 1% agarose gels prestained with ethidium bromide and run for 75 minutes at 115 V in 1× TB buffer (44.5 mM Tris–boric acid, pH 7.5) at room temperature.

tRNA Annealing Gel Shift Assay. Gel Shift Assay between NL4-3 WT, d21, and d22 5′-Leaders and $tRNA^{Lys3}$ were conducted at either 1:0 or 1:1 ratio in annealing buffer (10 mM Tris-HCl, pH 7.5, 10 mM NaCl, 1 mM $MgCl_2$, and 140 mM KCl). RNA was kept at a 20 μM concentration to ensure dimerization. To promote tRNA binding, samples were heat annealed in a thermocycler (Bio-Rad) at 94 °C for 5 min, 85 °C for 15 min, 75 °C for 15 min, and 65 °C for 60 min as previously described (25). 100 ng of 5'-Leader RNA were loaded onto 2% agarose gels pre-stained with ethidium bromide and run for 75 minutes at 115 V in 1× TB buffer (44.5 mM Tris–boric acid, pH 7.5) at room temperature.

Western blots. Transfection supernatants were normalized based on p24 and resuspended in RIPA buffer. Protein lysate recovery was quantified by BCA (ThermoFisher). Western blots were carried out as previously described (26). The same membrane was assayed for p24 and gp41. We used the following primary antibodies: murine monoclonal anti-p24 (Abcam, clone 39/5.4A, 1:2000 dilution) and human monoclonal anti-gp41 (AIDS reagent program, clone 246-D, 1:1000 dilution). After incubation over night at 4C, the membranes were washed three times and incubated for one hour with respective secondary antibodies: HRP-conjugated goat anti-murine IgG (Biolegend, 1:1000 dilution) and HRP-conjugated goat anti-human IgG (Invitrogen, 1:1000 dilution). Membranes were washed and imaged by ECL (ThermoFisher) on an iBright imager (ThermoFisher).

Transmission Electron Microscopy. Cells were transfected as described above in 35 mm tissue culture dishes (Falcon 3001). After 24 hours cells were rinsed with 37°C PBS for 1 min, and then fixed with 2% paraformaldehyde 2% glutaraldehyde (both EM grade) 100 mM Sorenson's phosphate and 5 mM MgCl2 pH 7.4 for 1hr at room temperature on a slow rocker. After a 30 min buffer rinse containing 3% sucrose, cells were post-fixed in 1% osmium tetroxide reduced with 0.8% potassium ferrocyanide 100 mM phosphate 5 mM MgCl2 at 4°C for 1hr in the dark. Samples were then rinsed with 100 mM maleate buffer 3% sucrose and *en-bloc* stained with 2% aqueous uranyl acetate (0.22 um filtered) in the same buffer, for 1 hr in the dark. Plates were dehydrated in a graded series of ethanol then infiltrated in Eponate 12 (Pella) overnight without catalyst. The next day cells were further embedded with fresh epon + 1.5% DMP-30 (catalyst) then cured at 37°C for three days, and further polymerized at 60°C overnight. Epon was removed from the plastic dish and 3 mm circles punched out and glued to epon blanks for sectioning. 60 nm thin compression free sections were obtained with a Diatome diamond knife (45 degree). Sections were picked up on 1x2 mm formvar coated copper slot grids (Polysciences), and further stained with uranyl acetate followed by lead citrate. Grids were examined on a Hitachi H-7600 TEM operating at 80Kv. Images were digitally captured with an AMT XR-80 (8 mega pixel) CCD camera.

Duplex quantification of total LTR copies and specific provirus. As previously described (9, 27, 28), we optimized a duplex digital PCR assay that selectively amplifies the integration site (*ADK*.d22 or *DNAJB14*.d21), together with all proviruses retaining the R-U5 LTR region

(irrespectively of its 5' or 3' location). To ensure that most partitions contain only a single LTR copy, we subjected genomic DNA to controlled physical shearing to yield fragments of ~6000 nucleotides or shorter (Figure S9). To do so, we used g-tubes (Covaris) following manufacturer's instructions (11000 rpm for 30 seconds, twice). Extent of shearing was tested by RPP30 as previously described (2). Due to the proximity of the integration site to the 5' R-U5 junction, >90% of proviruses of interest are detected as double-positive events (Figure 6B). To confirm the specificity of the assay, we duplexed the same integration site-specific assay with primers and probes targeting the 5'-L deletions unique to *ADK*.d22 and *DNAJB14*.d21. This second assay design yielded comparable frequencies of double-positive partitions (Figure 6B). This assay can be used to calculate the relative abundance of a provirus of interest among all infected cells. However, estimating the fraction of proviruses retaining both LTRs is challenging, and previous studies suggest that solo LTRs can represent a large fraction of the proviral landscape (9). Total LTR copies were quantified with a set of primers and probes annealing to the R-U5 region as previously described (29). To selectively quantify proviruses of interest, we designed primers surrounding the site of HIV-1 integration (27, 28). The probe was designed to anneal both to the provirus and its flanking human genome (Supplementary Table S5). Digital PCR was carried out with either the Biorad QX200 with cycling conditions as previously described (27) or the Qiagen Qiacuity with the following cycling parameters: 95°C for 2', and 40 cycles of 95°C for 15'' and 58°C for 30''; default parameters were used for the detection of positive partitions. Frequencies of LTR and integration site copies were calculated based on cell equivalent input by RPP30. The abundance of a provirus of interest relative to the total pool of infected cells was calculated as the percentage of LTR copies belonging to that provirus (integration site copies x2) over the total LTR copies.

T cell subset analysis. Cryopreserved PBMCs were thawed and rested for 4 hours in RPMI with 10% FBS. We incubated cells with FcgR block (BD Pharmingen) at room temperature for 10 minutes. Cells were stained, with a 30 minutes incubation on ice, with an APC-labelled antibody to CD3 (Biolegend; clone UCHT1), phycoerythrin (PE)-Cy7-labelled antibody to CD4 (Biolegend; clone RPA-T4), BV421-labelled antibody to CD45RA (BD Biosciences; clone HI100), PE-labelled antibody to CCR7 (Biolegend; clone G043H7) and PE-Cy5-labelled antibodies to CD14 (Thermo Fisher; clone 61D3), CD16 (Biolegend; clone 3G8) and CD20 (Biolegend; clone 2H7). Dead cells were excluded using propidium iodide. Cells stained with single fluorophore-labelled antibodies were used to set sorting gates. CD45RA expression was used to distinguish Naïve-like and terminally differentiated cells from memory cells. Central memory cells were distinguished from effector memory cells by the expression of CCR7. Naïve-like (Na), Central Memory (CM), Effector Memory (EM) and Effector Memory CD45RA positive (EMRA) subsets were sorted using either the Beckman Coulter MoFlo Legacy or XDP cell sorters. Sorted cell fractions were pelleted and stored at -80C until used for gDNA extraction with QIAamp DNA mini kit (Qiagen). TCR repertoires were obtained via Adaptive Biotechnologies (Seattle, WA) as previously described (27).

Supplementary References

1. Laird GM, Eisele EE, Rabi SA, Lai J, Chioma S, Blankson JN, et al. Rapid Quantification of the Latent Reservoir for HIV-1 Using a Viral Outgrowth Assay. *PLOS Pathogens.* 2013;9(5):e1003398.
2. Bruner KM, Wang Z, Simonetti FR, Bender AM, Kwon KJ, Sengupta S, et al. A quantitative approach for measuring the reservoir of latent HIV-1 proviruses. *Nature.* 2019;566(7742):120-5.
3. Simonetti FR, White JA, Tumiotto C, Ritter KD, Cai M, Gandhi RT, et al. Intact proviral DNA assay analysis of large cohorts of people with HIV provides a benchmark for the

frequency and composition of persistent proviral DNA. *Proc Natl Acad Sci U S A.* 2020;117(31):18692-700.
4. Coffin JM, and Hughes SH. Clonal Expansion of Infected CD4+ T Cells in People Living with HIV. *Viruses.* 2021;13(10).
5. Jiang C, Lian X, Gao C, Sun X, Einkauf KB, Chevalier JM, et al. Distinct viral reservoirs in individuals with spontaneous control of HIV-1. *Nature.* 2020;585(7824):261-7.
6. Laskey SB, Pohlmeyer CW, Bruner KM, and Siliciano RF. Evaluating Clonal Expansion of HIV-Infected Cells: Optimization of PCR Strategies to Predict Clonality. *PLoS Pathog.* 2016;12(8):e1005689.
7. Patro SC, Brandt LD, Bale MJ, Halvas EK, Joseph KW, Shao W, et al. Combined HIV-1 sequence and integration site analysis informs viral dynamics and allows reconstruction of replicating viral ancestors. *Proc Natl Acad Sci U S A.* 2019;116(51):25891-9.
8. Einkauf KB, Lee GQ, Gao C, Sharaf R, Sun X, Hua S, et al. Intact HIV-1 proviruses accumulate at distinct chromosomal positions during prolonged antiretroviral therapy. *J Clin Invest.* 2019;129(3):988-98.
9. Anderson EM, Simonetti FR, Gorelick RJ, Hill S, Gouzoulis MA, Bell J, et al. Dynamic Shifts in the HIV Proviral Landscape During Long Term Combination Antiretroviral Therapy: Implications for Persistence and Control of HIV Infections. *Viruses.* 2020;12(2).
10. Bacchus-Souffan C, Fitch M, Symons J, Abdel-Mohsen M, Reeves DB, Hoh R, et al. Relationship between CD4 T cell turnover, cellular differentiation and HIV persistence during ART. *PLoS Pathog.* 2021;17(1):e1009214.
11. Morcilla V, Bacchus-Souffan C, Fisher K, Horsburgh BA, Hiener B, Wang XQ, et al. HIV-1 Genomes Are Enriched in Memory CD4(+) T-Cells with Short Half-Lives. *mBio.* 2021;12(5):e0244721.
12. Coffin JM, Bale MJ, Wells D, Guo S, Luke B, Zerbato JM, et al. Integration in oncogenes plays only a minor role in determining the in vivo distribution of HIV integration sites before or during suppressive antiretroviral therapy. *PLoS Pathog.* 2021;17(4):e1009141.
13. Einkauf KB, Osborn MR, Gao C, Sun W, Sun X, Lian X, et al. Parallel analysis of transcription, integration, and sequence of single HIV-1 proviruses. *Cell.* 2022;185(2):266-82 e15.
14. Bushman FD. Retroviral Insertional Mutagenesis in Humans: Evidence for Four Genetic Mechanisms Promoting Expansion of Cell Clones. *Mol Ther.* 2020;28(2):352-6.
15. Miron M, Meng W, Rosenfeld AM, Dvorkin S, Poon MML, Lam N, et al. Maintenance of the human memory T cell repertoire by subset and tissue site. *Genome Med.* 2021;13(1):100.
16. Grossman Z, Singh NJ, Simonetti FR, Lederman MM, Douek DC, Deeks SG, et al. 'Rinse and Replace': Boosting T Cell Turnover To Reduce HIV-1 Reservoirs. *Trends Immunol.* 2020;41(6):466-80.
17. Bertagnolli LN, Varriale J, Sweet S, Brockhurst J, Simonetti FR, White J, et al. Autologous IgG antibodies block outgrowth of a substantial but variable fraction of viruses in the latent reservoir for HIV-1. *Proc Natl Acad Sci U S A.* 2020;117(50):32066-77.
18. Williams KL, Stumpf M, Naiman NE, Ding S, Garrett M, Gobillot T, et al. Identification of HIV gp41-specific antibodies that mediate killing of infected cells. *PLoS Pathog.* 2019;15(2):e1007572.
19. Rajashekar JK, Richard J, Beloor J, Prevost J, Anand SP, Beaudoin-Bussieres G, et al. Modulating HIV-1 envelope glycoprotein conformation to decrease the HIV-1 reservoir. *Cell Host Microbe.* 2021;29(6):904-16 e6.
20. Keane SC, Van V, Frank HM, Sciandra CA, McCowin S, Santos J, et al. NMR detection of intermolecular interaction sites in the dimeric 5'-leader of the HIV-1 genome. *Proc Natl Acad Sci U S A.* 2016;113(46):13033-8.
21. Tran T, Liu Y, Marchant J, Monti S, Seu M, Zaki J, et al. Conserved determinants of lentiviral genome dimerization. *Retrovirology.* 2015;12:83.

22. Lee BM, De Guzman RN, Turner BG, Tjandra N, and Summers MF. Dynamical behavior of the HIV-1 nucleocapsid protein. *J Mol Biol.* 1998;279:633-49.
23. Ding P, Kharytonchyk S, Waller A, Mbaekwe U, Basappa S, Kuo N, et al. Identification of the initial nucleocapsid recognition element in the HIV-1 RNA packaging signal. *Proc Natl Acad Sci U S A.* 2020;117(30):17737-46.
24. Keane SC, Heng X, Lu K, Kharytonchyk S, Ramakrishnan V, Carter G, et al. RNA structure. Structure of the HIV-1 RNA packaging signal. *Science.* 2015;348(6237):917-21.
25. Gremminger T, Song Z, Ji J, Foster A, Weng K, and Heng X. Extended interactions between HIV-1 viral RNA and tRNALys3 are important to maintain viral RNA integrity. *International Journal of Molecular Sciences.* 2020;22(1):58.
26. Kirchherr JL, Hamilton J, Lu X, Gnanakaran S, Muldoon M, Daniels M, et al. Identification of amino acid substitutions associated with neutralization phenotype in the human immunodeficiency virus type-1 subtype C gp120. *Virology.* 2011;409(2):163-74.
27. Simonetti FR, Zhang H, Soroosh GP, Duan J, Rhodehouse K, Hill AL, et al. Antigen-driven clonal selection shapes the persistence of HIV-1-infected CD4+ T cells in vivo. *J Clin Invest.* 2021;131(3).
28. Brandt LD, Guo S, Joseph KW, Jacobs JL, Naqvi A, Coffin JM, et al. Tracking HIV-1-Infected Cell Clones Using Integration Site-Specific qPCR. *Viruses.* 2021;13(7).
29. Anderson EM, and Maldarelli F. The role of integration and clonal expansion in HIV infection: live long and prosper. *Retrovirology.* 2018;15(1):71.

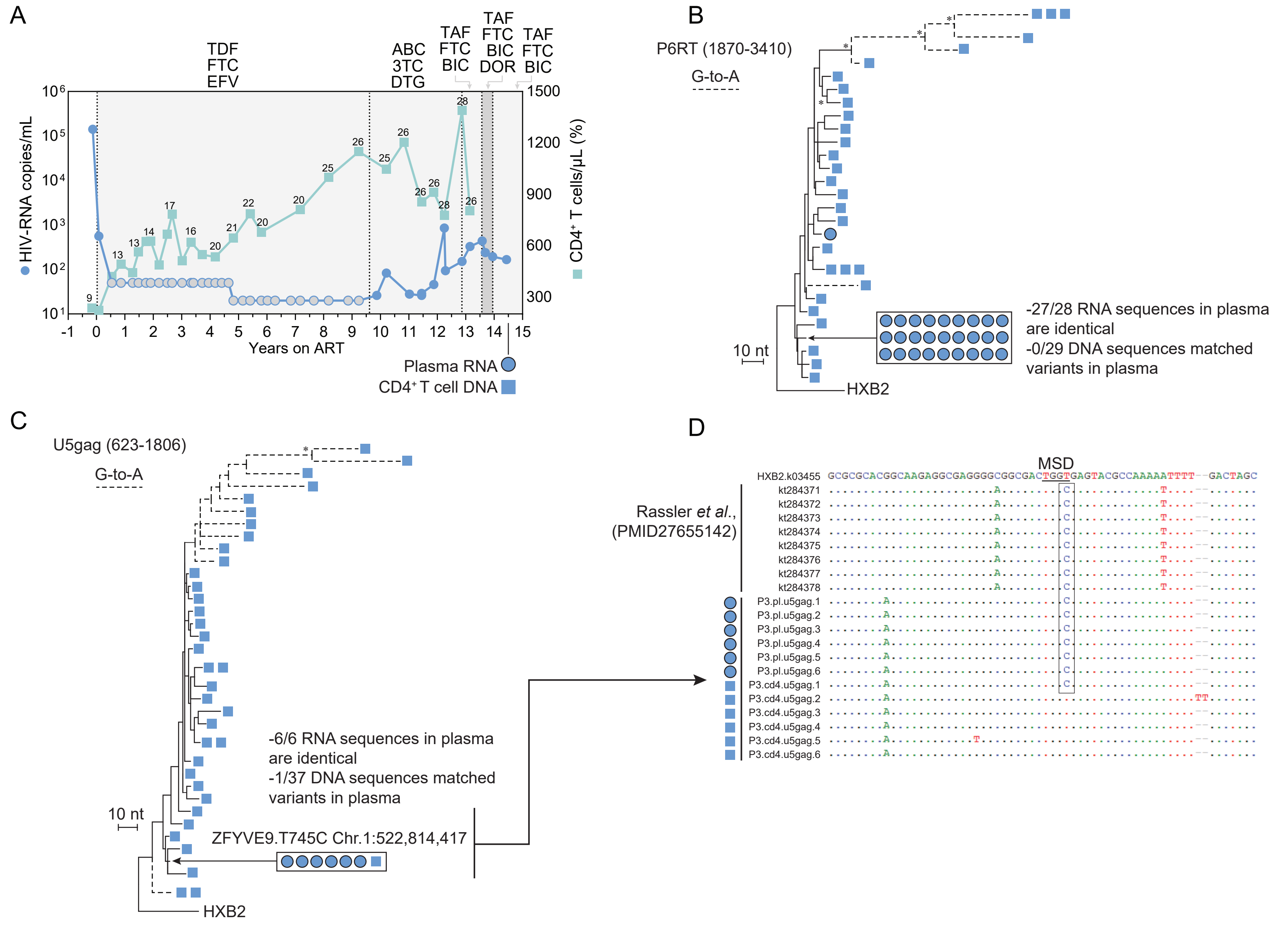

Supplementary Figure S1. Clinical history of Study Participant P3 with nonsuppressible viremia (NSV), and analysis of HIV-1 populations in plasma and CD4⁺ T cells. (**A**) Plasma HIV-1 RNA and CD4⁺ T cell counts over time for P3; grey circles indicate values below the limit of quantification of the clinical assay; numbers above squares indicate CD4⁺ T cell percentage; light grey areas indicate standard ART, dark grey areas indicate ART intensification; ART regimens are indicated above the graph; time points and samples analyzed in this study are shown below the graph. (**B**) Maximum likelihood tree analysis of *P6*-RT single genome sequences from P3; dashed branch lines indicate sequences with APOBEC3G/F-induced hypermutation; tree nodes with bootstrap values above 80 are marked by star symbols; identical sequences matching proviruses with integration and full genome data are highligthed in boxes. (**C**) Maximum likelihood tree analysis of U5-*gag* single genome sequences from P3; chromosomal location is indicated above boxed area. (**D**) 5'-L sequences of reisdual plasma virus published by Rassler *et al*. aligned to the predominant plasma clone found in P3; sequences are aligned to HXB2. 3TC lamivudine, ABC abacavir, FTC emtricitabine, TDF tenofovir disoproxil fumarate, TAF tenofovir alanfenamide, DTG dolutegravir, BIC bictegravir, EVF efavirenz, DOR doravirine.

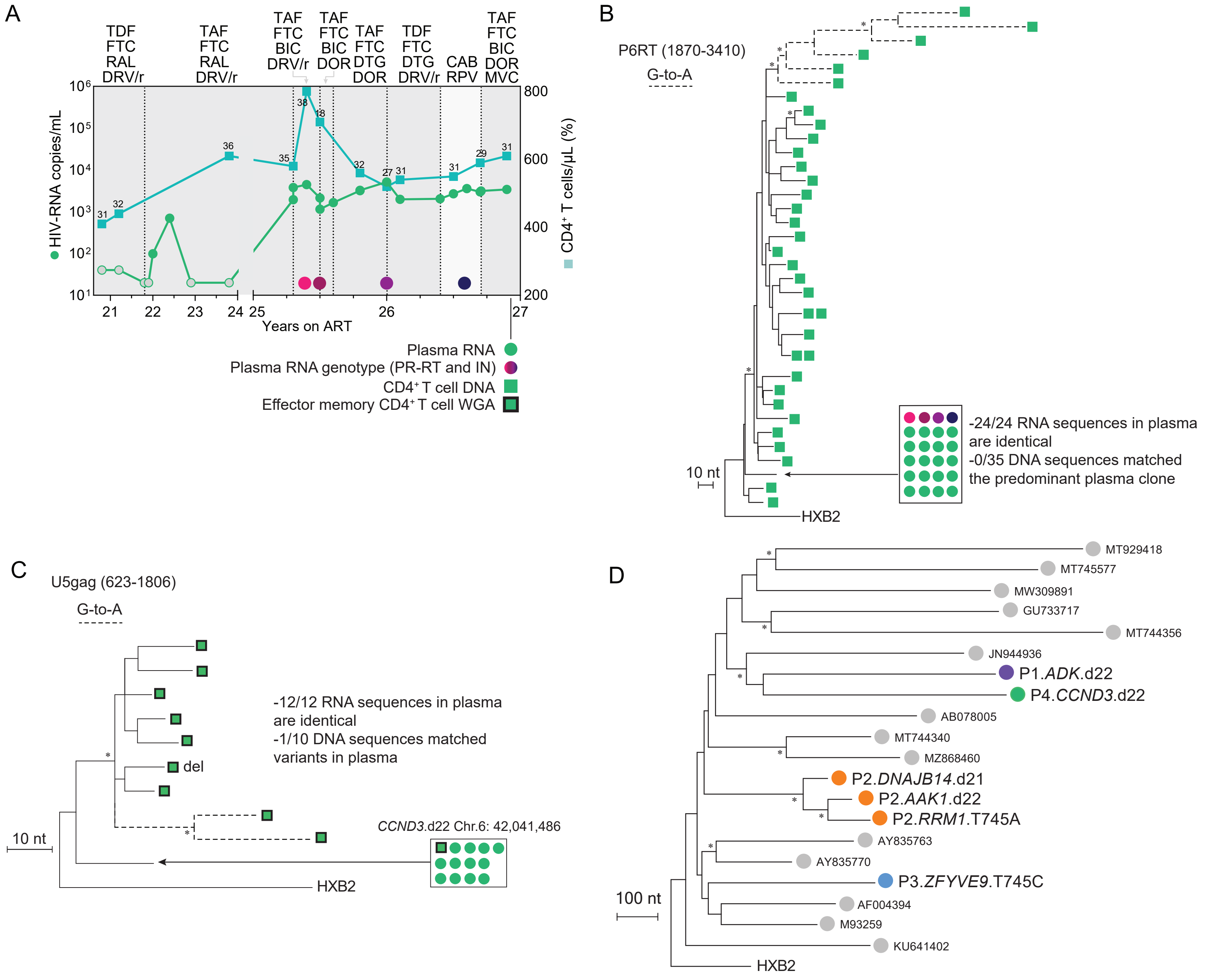

Supplementary Figure S2. Clinical history of study participant P4 with nonsuppressible viremia (NSV), and analysis of HIV-1 populations in plasma and CD4+ T cells. (**A**) Plasma HIV-1 RNA and CD4+ T cell counts over time for P4; grey circles indicate values below the limit of quantification of the clinical assay; numbers above squares indicate CD4+ T cell percentage; light grey areas indicate standard ART, dark grey areas indicate ART intensification; ART regimens are indicated above the graph; time points and samples analyzed in this study are shown below the graph. (**B**) Maximum likelihood tree analysis of P6-RT single genome sequences from P4; dashed branch lines indicate sequences with APOBEC3G/F-induced hypermutation; tree nodes with bootstrap values above 75 are marked by star symbols; identical sequences matching proviruses with integration and full genome data are highligthed in boxes. (**C**) Maximum likelihood tree analysis of U5-*gag* single genome sequences from P4; chromosomal location is indicated above boxed area. (**D**) maximum likelihood tree analysis of near full length genomes of the proviruses causing viremia from all participants together with 15 subtype B references. FTC emtricitabine, TDF tenofovir disoproxil fumarate, TAF tenofovir alanfenamide, RAL raltegravir, DTG dolutegravir, BIC bictegravir, CAB cabotegravir, DOR doravirine, RPV rilpivirine, DRV/r darunavir/ritonavir, MVC, maraviroc.

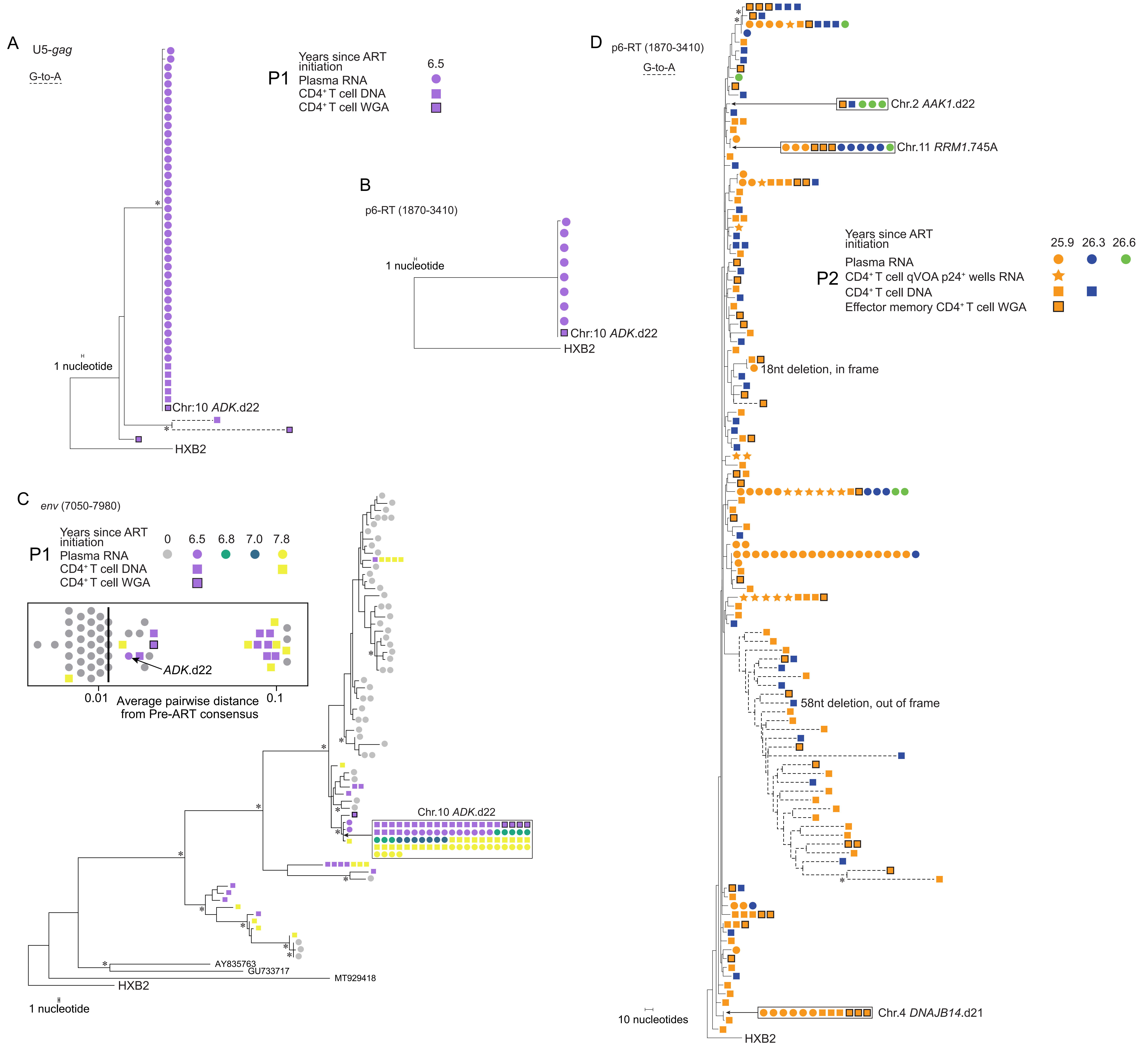

Supplementary Figure S3. Additional HIV-1 sequence analyses from P1 and P3 related to Figure 1. (**A**) Maximum likelihood tree analysis of U5-*gag* single genome sequences from P1; dashed branch lines indicate sequences with APOBEC3G/F-induced hypermutation; tree nodes with bootstrap values above 75 are marked by star symbols; HBX2 reference sequence is used as outgroup; timepoints and sample types are color-coded as indicated in the legend. (**B**) Maximum likelihood tree analysis of P6-RT single genome sequences from P3. (**C**) Maximum likelihood tree analysis of *env* single genome sequences from P1 (related to Figure 1C) including RNA sequences found in plasma at the time of ART initiation (grey circles); hypermutated sequences are excluded from this analysis; boxed scatter plot shows average pairwise distances from the consensus of all pre-ART plasma sequences; identical sequences are sampled only once; the black vertical bar indicates median distance; (**D**) Complete maximum likelihood tree analysis of P6-RT single genome sequences from P2, related to Figure 1D; proviruses of interest with 5'-Leader deletion are highilighted by black boxes.

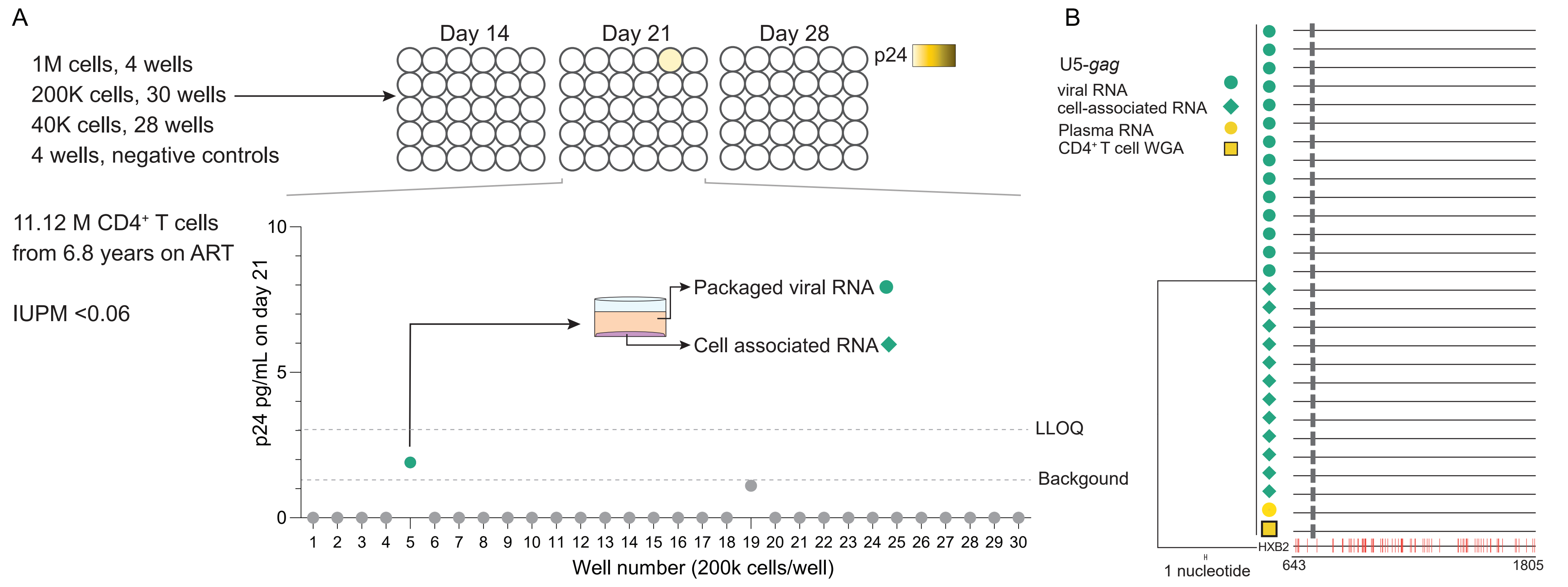

Supplementary Figure S4. Quantitative viral outgrowth assay from P1 shows lack of exponential replication of predonimant plasma clone.
(**A**) Experimental set up of viral outgrowth assay from total CD4⁺ T cells from P1; the graph show p24 levels in each of the wells with 200k cells at day 21; RNA was extracted from cells and pelleted viral particles from the supernatant of well 5, which had low p24 signal; LLOQ, lower limit of quantification, is based on the lowest values of the standard curve. (**B**) Neighbor-joining tree of U5-*gag* single genome sequences recovered from QVOA well number 5 aligned to the predominant vairant found in plasma; HXB2 reference sequences is used as outgroup; highlighter plot on the right shows mutations relative to predominant plasma clone in P1; 22-nucleotide deletion is indicated in grey.

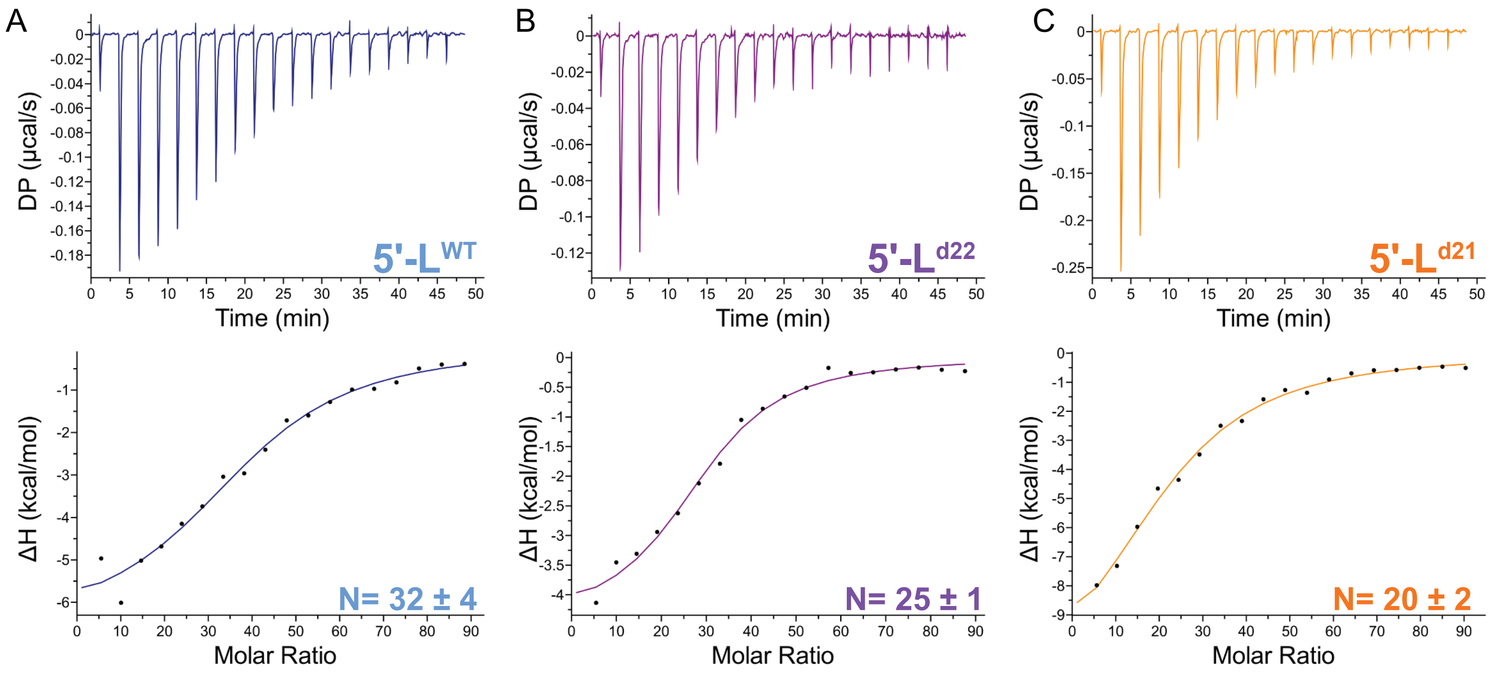

Supplementary Figure S5. 5'-Leader deletions cause a modest decrease in the number of nucleocapsid binding sites. (**A**, **B**, **C**) ITC isotherms of WT (**A**), d22 (**B**), and d21 (**C**) truncated leader constructs with high ratios of NC to reach saturation. Fitted N values are indicated with standard deviation of values across four replicates. The fitted Kd values for WT< d21, and d22 constructs were 1.8, 3.9 and 1.7 µM, respectively; however, these values reflect a range of affinities for the >20 individual NC bindings sites. See Table S3 for detailed statistics.

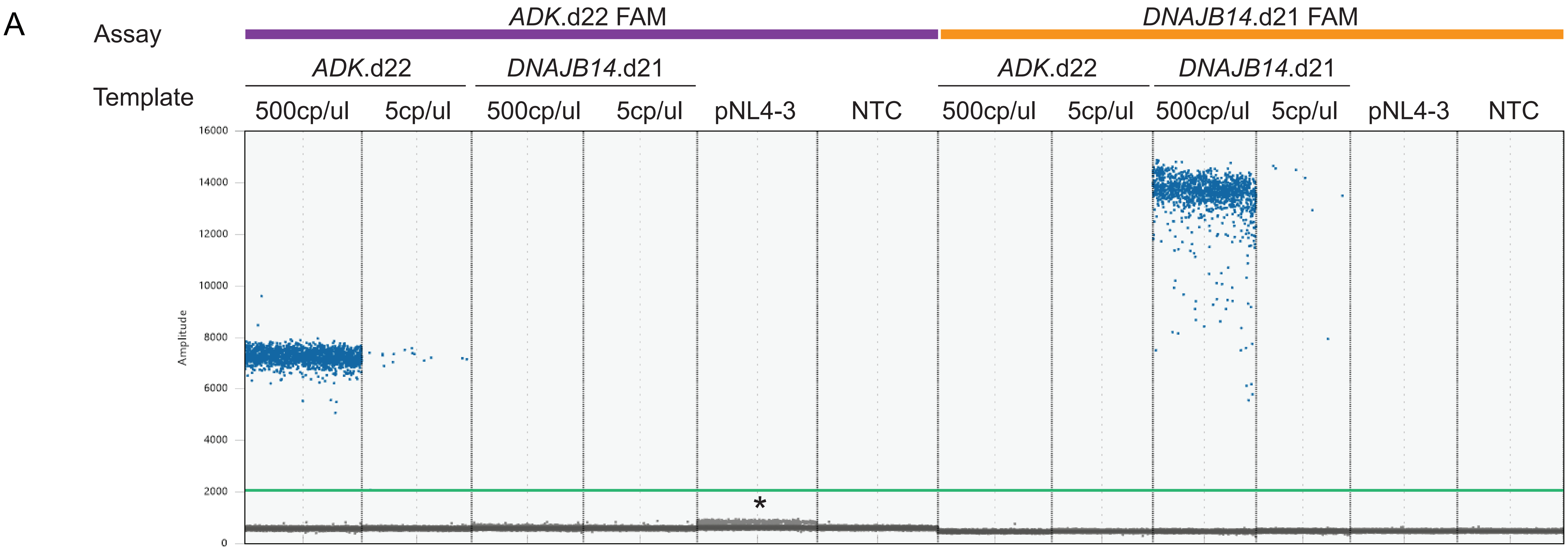

Supplementry Figure S6. Validation of digital PCR assays that selectively amplify proviruses of interest, related to Figure 4. (**A**) Results of droplet digital PCR using primers and probe sets to amplify sequences across 5'-L deletions of proviruses of interest. To assess specificity, each assay was tested with synthetic double strand DNA identical to *ADK*.d22 or *DNAJB14*.d21, plasmid containing wild type NL4-3, or water (NTC). The green bar indicates the threshold for positive partitions. The star symbol indicates dim partitions from pNL4-3 non-spefic signal, correctly classified as negative for *ADK*.d22.

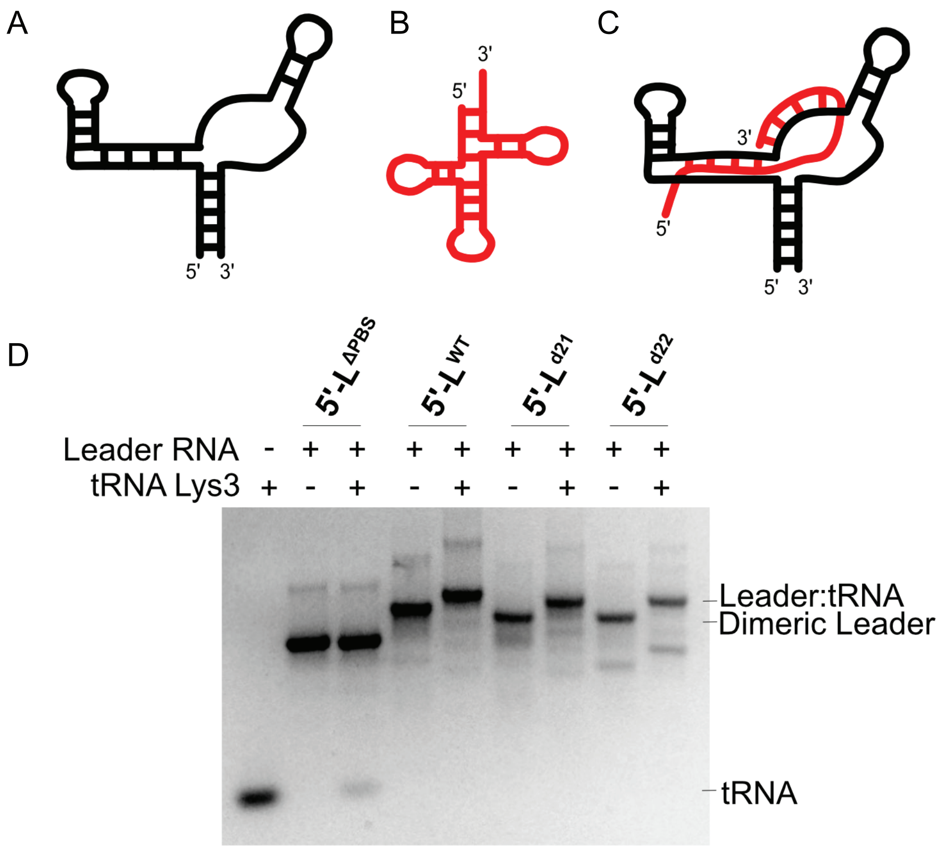

Supplementary Figure S7. Deletions do no alter tRNA binding propensity to the 5'-Leader. (**A**) Proposed PBS secondary structure. (**B**) tRNA Lys3 secondary structure. (**C**) Model of tRNA binding to PBS in an extended conformation proposed by Gremminger et al. (25) (**D**) EMSA of 5'-Leader constructs in presence and absence of tRNA. Lane 1 is tRNA with increase material loaded compared to subsequent lanes to appropriately visualize tRNA on the gel. Lane 2, 4, 6, 8 are 5'-Leader constructs with deletion of PBS, wildtype, d21 mutation, and d22 mutation respectively without tRNA, while Lanes 3, 5, 7, 9 are in presence of $tRNA^{Lys3}$.

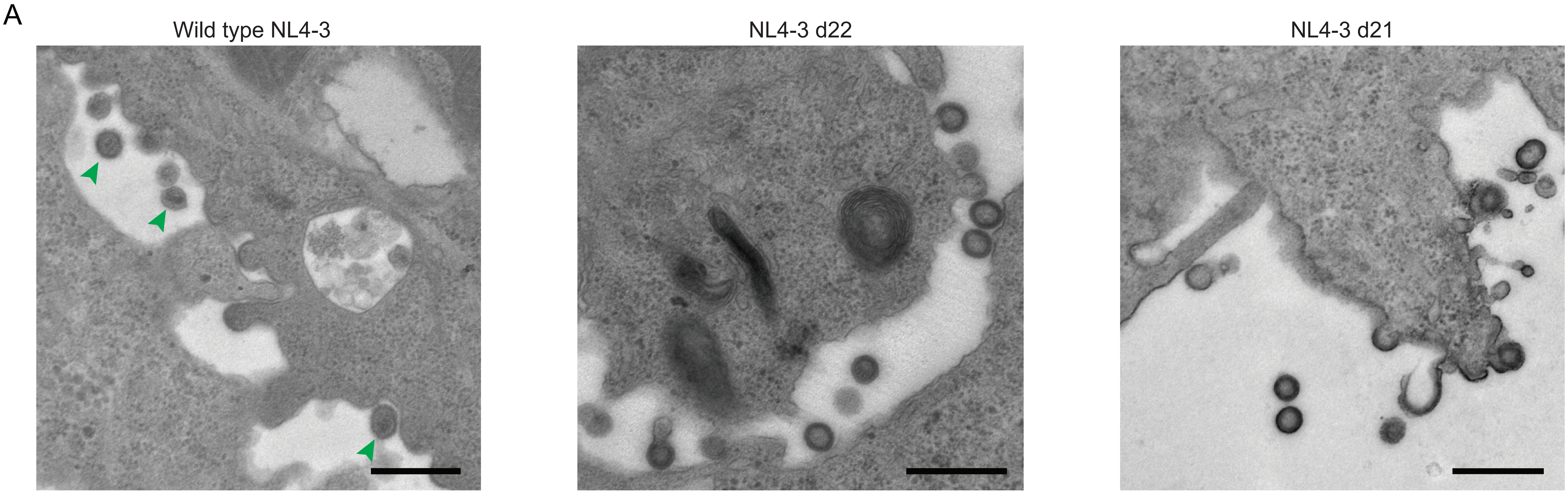

Supplementary Figure S8. Production of viral particles upon transfection with 5'-Leader defective NL4-3. (**A**) 293T cells were transfected with 5'-Leader wild type or mutant NL4-3 plasmids; at 24 hours cells were rinsed with warm PBS and fixed for thin-section electron microscopy. Black bars indicate 500nm. Green arrow heads indicate mature virions with conical morphology of the capsid.

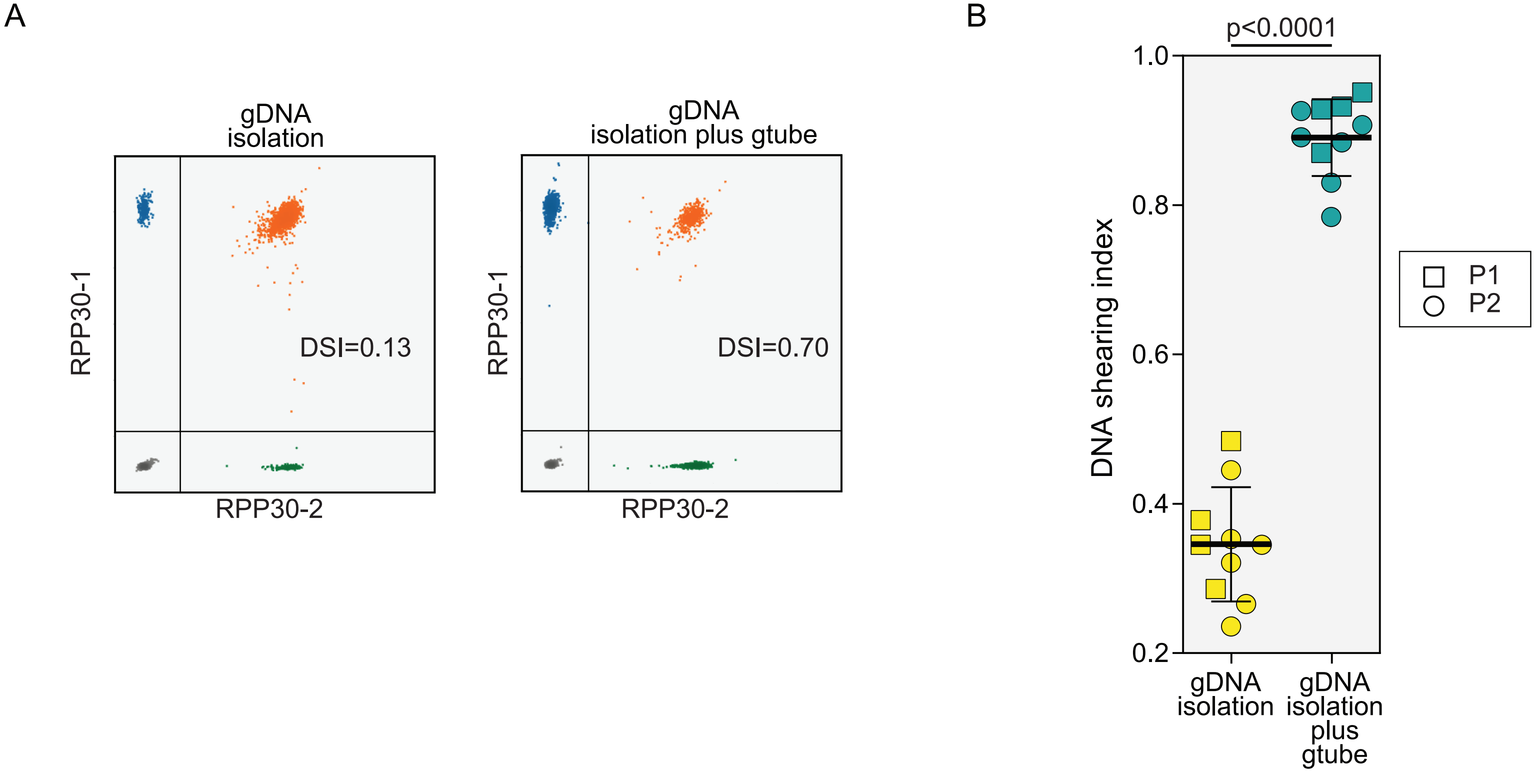

Supplementary Figure S9. Impact of controlled shearing on genomic DNA used in integration site-specific dPCR assays. (**A**) Droplet digital PCR 2D plots showing RPP30 amplification before and after controlled DNA shearing; DSI, DNA shearing index. (**B**) Controlled shearing leads to singificant increase in DSI values; symbols indicate DSI values of matched DNA samples used in Figures 6 and 7, before and after physical shearing with g-tubes (see methods); statistical significancw was tested by parametric paired t-test.

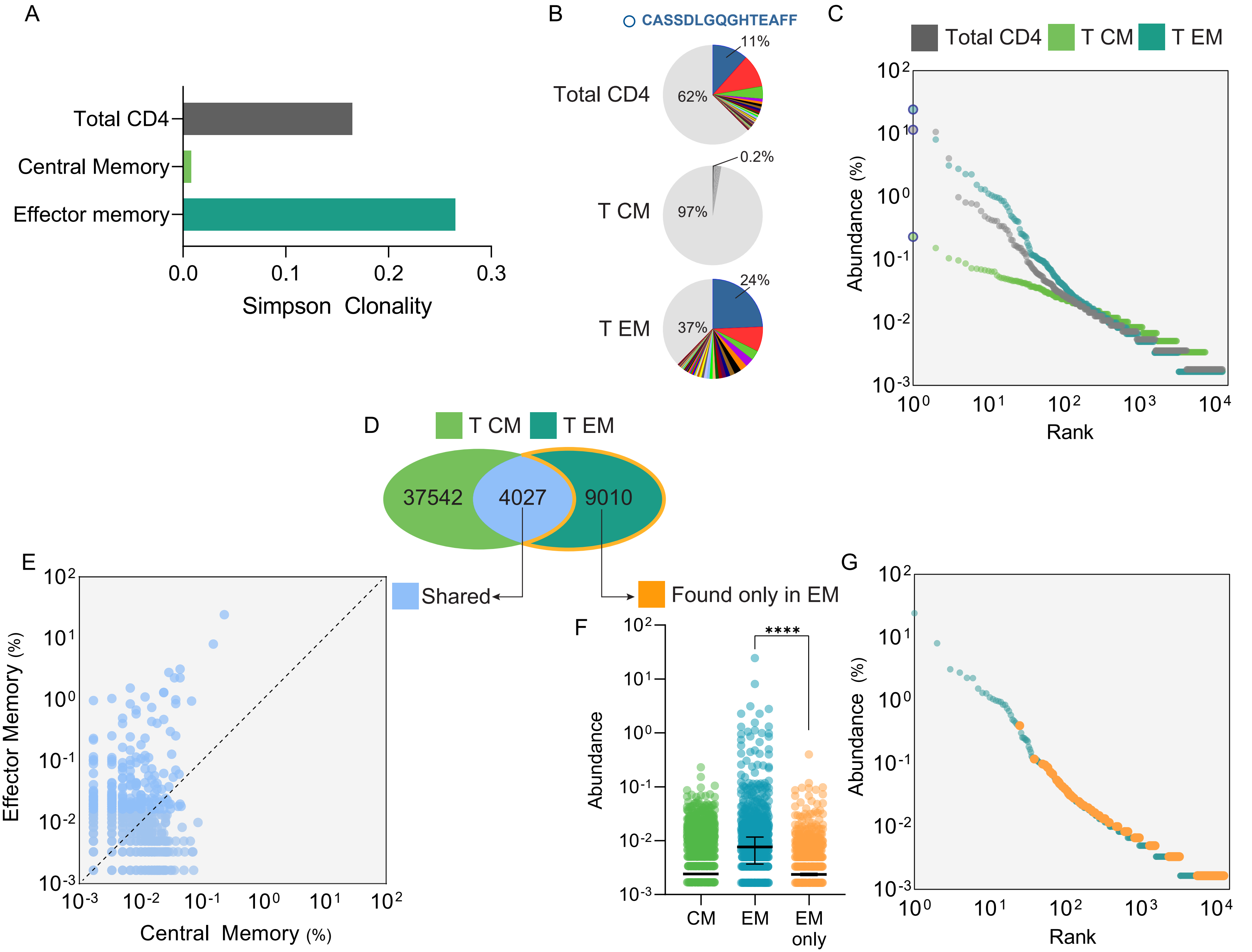

Supplementary Figure S10. TCRβ sequence analysis of total, Central, and Effector memory CD4⁺ T cell subsets from P1. (**A**) Higher Simpson Clonality index of TCRβ sequences in EM than in CM cells. (**B**) Relative abundance (%) of the 50 most expanded CDR3 aminoacid sequences across the three groups of cells; sequences below the top 50 rank are grouped together in grey; the percentage of the most expanded CDR3 sequence CASS-DLGQGHTEAFF is shown above each pie chart. (**C**) Rank-aundance plot of TCRβ sequences; steeper curves indicate higher clonal dominance; the most abundant sequences is highlighted in blue. (**D**) Analysis of repertoire overlap between CM and EM cells; numbers indicate unique TCRβ sequences; light blue represents shared sequences while orange indicates those unique to EM cells. (**E**) Analisys of differential abundance of TCRβ sequences shared between CM and EM cells. (**F**) Cells found only in EM compartment have a lower mean abundance than total EM cells; errora bars indicate 95% confidence interval. (**G**) Rank-aundance plot of TCRβ sequences from cell found only in EM cells (orange) versus total EM cells.

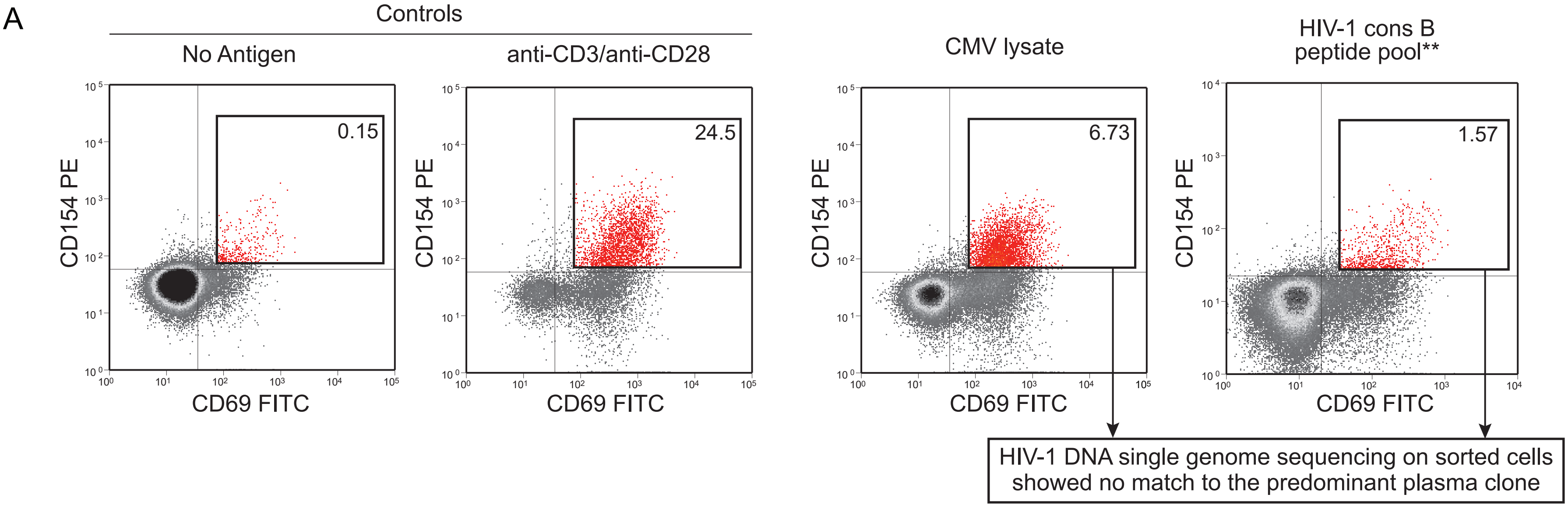

Supplementary Figure S11. Isolation of antigen-responding CD4⁺ T cells from PBMCs in P1.
(**A**) Stimulation of CD8-depleted PBMCs for 16 hours with CMV and HIV-1 Gag antigens leads to upregulation of activation markers CD154 and CD69; left panels show negative and positive controls carried out with no antigen and anti-CD3/anti-CD28 antibody-coated beads, respectively; numbers in gates indicate percentage of doulbe positive events; **cells stimulated with HIV-1 Gag were analyzed and sorted on a Backman Coulter MoFlo instrument, while all other conditions were run on a XDP instrument.

Characteristics	P1	P2	P3	P4	Median[d]
Sex	Male	Male	Female	Male	
Age (y)	63	60	58	60	60
Race	African American	Caucasian/White	African American	Caucasian/White	
Years since diagnosis	30	31	15	32	31
Years on ART	7.8	26.4	14.5	27	20
$CD4^+$ T cell count nadir (cells/mm^3)	454	197	221	na	
$CD4^+$ T cell count, last (cells/mm^3)	828	793	803	610	798
HIV-1 RNA, setpoint (copies/mL)	8771	na[c]	141667	na	
HIV-1 RNA, last (copies/mL)[a]	58	20	167	3400	113
Years with detectable viremia	5	11	5	5	5
ART regimen, last[b]	TAF,FTC,BIC	TAF,FTC,BIC,FTR	TAF,FTC,BIC	TAF, FTC, BIC, DOR, MVC	
Infectious units per million (QVOA)	<0.06	15 (10-21)	na	na	
Intact proviruses/10^6 $CD4^+$ T cells (IPDA)	3.5	311	161	58	110
HLA-B	53:01, 57:03	44:02	44:03	44:03, 57:02	

Supplementary Table S1. Participant characteristics. a, measured with limit of detection of 20 copies/mL; b, TAF tenofovir alafenamide fumarate, FTC emtricitabine, BIC bictegravir, FTR fostemsavir, DOR doravirine, MVC maraviroc; c, not available. d, median values were calculated when available for all 4 participants.

Sample		Provirus						Host gene					
**Participant ID**	**Provirus ID**	**Chromosome**	**Position[a]**	**5 nt duplication**	**Host junctions recovered**	**Strand**	**Orientation relative to gene**	**Gene symbol**	**Gene ID (NCBI)**	**Gene transcription orientation**	**Provirus location[b]**	**Expression levels in effector memory CD4^{+} cells[c]**	**Pathway[c]**
**P1**	p1.ADK.d22	10	74192042	GTTAC	5', 3'	-	opposite	*ADK*	132	+	intron 1/9	medium	Purine metabolism
**P2**	p2.AAK1.d22	2	69478200	CTATG	5', 3'	+	opposite	*AAK1*	22848	-	intron 17/21	medium	Clathrin-mediated endocytosis, Vesicle-mediated transport
	p2.DNAJB14.d21	4	99910773	TTCAT	5', 3'	+	opposite	*DNAJB14*	79982	-	intron 3/7	medium	Hsp70 protein binding
	p2.RRM1.T745A	11	4117665	GCCAC	5', 3'	-	opposite	*RRM1*	6240	+	intron 7/18 (1/12)	medium	Pyrimidine and purine nucleotides *de novo* biosynthesis
**P3**	p3.ZFYVE9.T745C	1	522814417	GGCTC	5', 3'	-	opposite	*ZFYVE9*	9372	+	intron 9/18	vey low	TGF-beta receptor signaling
**P4**	p4.CCND3.d22	6	42041486	GTAAC	5', 3'	+	opposite	*CCND3*	896	-	intron 1/3	high	Cyclin-dependent protein serine/threonine kinase activity, G1/S transition

a Number refers to the third nucleotide of the 5nt duplication, using GRCH38/hg38 assembly
b Numbers in parentheses refer to alternative isoforms
c Based on Immune Cell Atlas (http://immunecellatlas.net/) population RNA-seq, normalized expression values (0-5 trace, very low 5-20, low 20-80, 80-800 medium , 800-8000 high)
d Based on Gene Onthology Project (http://geneontology.org/)

Supplementary Table S2. Integration site analysis of defective proviruses causing NSV

RNA Construct	[Nucleocapsid] (μM)	[Dimer] (μM)	N (sites)	N Error (sites)	Kd (μM)	Kd Error (μM)	ΔH (kcal/mol)	ΔG (kcal/mol)	-TΔS (kcal/mol)	Offset (kcal/mol)	Red. Chi-Sqr. (kcal/mol)²
**NL4-3 WT**	225	0.49	33.7	1.4	1.63	0.55	-5.02	-7.90	-2.87	0.11	4.10E-02
			28.9	1.0	1.21	0.39	-4.87	-8.07	-3.20	-0.13	3.20E-02
			36.2	1.3	1.62	0.49	-4.76	-7.90	-3.14	-0.11	3.00E-02
			28.0	2.0	2.75	1.32	-6.54	-7.59	-1.05	0.07	8.10E-02
**NL4-3 d22**	225	0.50	24.9	1.0	1.14	0.35	-3.60	-8.11	-4.50	0.08	1.80E-02
			25.3	1.3	1.19	0.49	-3.28	-8.08	-4.81	0.06	2.60E-02
			22.8	2.1	2.75	1.47	-4.71	-7.59	-2.88	0.16	4.60E-02
			25.3	1.1	1.67	0.53	-3.50	-7.88	-4.38	0.05	1.50E-02
**NL4-3 d21**	225	0.48	22.9	1.0	2.95	0.68	-9.55	-7.55	2.00	0.28	3.20E-02
			18.6	1.3	3.76	1.02	-11.50	-7.40	4.06	0.26	3.90E-02
			17.9	1.7	5.68	1.49	-13.30	-7.16	6.10	0.38	2.40E-02
			19.5	1.2	3.23	0.84	-10.70	-7.49	3.21	0.12	4.00E-02

Supplementary Table S3. ITC Isotherm Fitting Data of NC to NL4-3 WT, d21, d22 to full saturation. Data was fit using the MicroCal PEAQ-ITC analysis software provided by Malvern. A converged fit was achieved using the Levenberg-Marquardt algorithm. Errors of fit are indicated.

Participant ID	A	B	C
P1	34:02, 74:01	57:03, 53:01	04:01, 07:01
P2	02:01, 32:01	44:02	05:01, 07:04
P3	02:01, 30:02	44:03	04:01, 14:03
P4	03:01, 29:02	57:02, 44:03	16:01, 18:01

P1	region	relevant MHC	expected	observed	impact	WT?	B*57	B*57 WT?
ADK.d22	gag	B*53:01	TPQDLNTML	TPQDLNTML		1		
		B*53:01	ETINEEAAEW	ETINEEAAEW		1		
		B*57:03	AISPRTLNAW	**PL**SPRTLNAW	A1P, I2L documented escape	0	1	
		B*57:03	KAFSPEVIPMF{S}	KAFSPEVIPMF**{T}**	S+1T literature escape, reduced fitness	0	1	
		B*57:03	TSTLQEQIGW	TS**N**LQEQI**A**W	T3N documented escape, reduced fitness	0	1	
		B*57:03	QASQEVKNW	QASQ**D**VKNW	Weak Binder	0	1	
	pol	B*57:03	STTVKAACWW	S**NV**VKAACWW	T2N documented escape	0	1	
		B*57:03	PIVLPEKDSW	PI**L**LPEKDSW	Weak Binder	0	1	
		A*74:01	SQIYPGIKVR	SQIYPGIKVR		1		
	vif	B*57:03	ISRKAKGWF	ISRKAK**N**W**L**	Weak Binder	0	1	
	vpr	B*57:03	AVRHFPRIW	AVRHFPRIW		1	1	1
	rev	B*57:03	LRAVRIIKI	L**K**AVR**L**IK**F**	I9F diminished response	0	1	
	env	C*04:01	SFNCGGEFF	**T**FNCGGEFF	S1T documented escape	0		
		C*04:01	QFRNKTIVF	QF--**HGPIA**F	Non Binder	0		
		C*07:01	VYYGVPVWKEA	VYYGVPVWKEA		1		
	nef	B*57:03	KAAFDLSFF	K**G**A**V**DLS**H**F	T2G diminished response	0	1	
		B*57:03	HTQGYFPDW	HTQGY**L**PDW	Strong binder	1	1	1
		C*07:1	KRQDILDLWVY	KRQDILDLWV**H**	Binding unchanged	1		
		C*07	RYPLTFGWCY	RYPLTFGWC**F**	Y10F binding unchanged	1		
total			19			42.1	11	18.2

P2	region	relevant MHC	expected	observed	impact	WT?
AAK1.d22	gag	A*02:01	SLYNTVATL	SLYNTVATL		1
		A*02:01	TLNAWVKVV	TLNAWVKVV		1
		A*02:01	MTSNPPIPV	MTSNPPIPV		1
		A*02:01	VLAEAMSQV	VLAEAMSQV		1
		B*44:02	AEQASQDVKNW	AEQ**CT**QEVKNW	Strong binder	1
	pol	A*02:01	KMIGGIGGFI	KMIGGIGGFI		1
		A*02:01	VLVGPTPVNI	VLVGPTPVNI		1
		A*02:01	ALVEICTEM	ALVEICTEM		1
		A*02:01	VIYQYMDDLYV	VIYQYMDDLYV		1
		A*02:01	ILKEPVHGV	ILKEPVHGV		1
		A*02:01	ALQDSGLEV	ALQDSGLEV		1
		A*32:01	PIQKETWETW	PIQ**R**ETW**D**TW	Non binder	0
		B*44:02	EEMNLPGRW	E**D**M**T**LPGRW	E2D inferred/documented escape	0
		B*44:02	QEEHERYHSNW	Q**DD**HEKYHSNW	Non binder	0
		C*05:01	HTDNGSNF	HTDNGSNF		1
	vpr	A*02:01	RILQQLLFI	RILQQLLFI		1
	rev	B*44:02	EELLKTVRL	EEL**I**KTVRL	L4I diminished response, escape	0
		C*05:01	SAEPVPLQL	**P**AEPVPLQL	Non binder	0
	env	A*02:01	KLTPLCVTL	KLTPLCVTL		1
		A*02:01	TLSQIVTKL	TL**D**QI**FK**KL	Non binder	0
		A*02:01	SLLNATDIAV	SLLNATAIAV		1
		A*02:01	RVIEVAQRV	RIIEV**L**QR**A**	Weak binder	0
		A*32:01	RIKQIINMW	RIKQIIN**L**W	Strong binder	1
		B*44:02	AENLWVTVY	**T**E**K**LWVTVY	A1T, N3K documented escape	0
		C*07:04	VYYGVPVWKEA	VYYGVPVWKEA		1
	nef	A*02:01	LEWRFDSTL	L**V**WRFDS**R**L	Non binder	0
		A*02:01	AFHHVAREL	AFHH**M**AREL	Non binder	0
		C*07:04	KRQDILDLWVY	KRQDILDLWVY		1
		C*07:04	RYPLTFGWCY	RYPLTFGWC**F**	Y10F binding unchanged	1
total			29			65.5

P2	region	relevant MHC	expected	observed	impact	WT?
DNAJB14.d21	gag	A*02:01	SLYNTVATL	SLYNTVATL		1
		A*02:01	TLNAWVKVV	TLNAWVKVV		1
		A*02:01	MTSNPPIPV	MTSNPPIPV		1
		A*02:01	VLAEAMSQV	VLAEAMSQV		1
		B*44:02	AEQASQDVKNW	AEQ**CT**QEVKNW	Strong binder	1
	pol	A*02:01	KMIGGIGGFI	KMIGGIGGFI		1
		A*02:01	VLVGPTPVNI	VLVGPTPVNI		1
		A*02:01	ALVEICTEM	ALVEICTEM		1
		A*02:01	VIYQYMDDLYV	VIYQYMDDLYV		1
		A*02:01	ILKEPVHGV	ILKEPVHGV		1
		A*02:01	ALQDSGLEV	ALQDSGLEV		1
		A*32:01	PIQKETWETW	PIQ**R**ETWETW	Weak binder	0
		B*44:02	EEMNLPGRW	E**D**M**T**LPGRW	E2D inferred/documented escape	0
		B*44:02	QEEHERYHSNW	Q**DD**HEKYHSNW	Non binder	0
		C*05:01	HTDNGSNF	HTDNG**G**NF	Strong binder	1
	vpr	A*02	AIIRILQQL	AIIRILQQL		1
	rev	B*44:02	EELLKTVRL	EELL**Q**TVRL	K5Q documented escape	0
		C*05:01	SAEPVPLQL	**P**AEPVPLQL	Non binder	0
	env	A*02:01	KLTPLCVTL	KLTPLCVTL		1
		A*02:01	TLSQIVTKL	TL**D**QI**FK**KL	Non binder	0
		A*02:01	SLLNATDIAV	SLLNATAIAV		1
		A*02:01	RVIEVAQRV	RIIEV**L**QR**A**	Weak binder	0
		A*32:01	RIKQIINMW	RIKQIIN**L**W	Strong binder	1
		B*44:02	AENLWVTVY	**T**E**K**LWVTVY	A1T, N3K documented escape	0
		C*07:04	VYYGVPVWKEA	VYYGVPVWKEA		1
	nef	A*02:01	LEWRFDSTL	L**V**WRFDS**R**L	Non binder	0
		A*02:01	AFHHVAREL	AFHH**M**AREL	Non binder	0
		C*07:04	KRQDILDLWVY	KRQDILDLWVY		1
		C*07:04	RYPLTFGWCY	RYPLTFGWC**F**	Y10F binding unchanged	1
total			29			65.5

P2	region	relevant MHC	expected	observed	impact	WT?
RRM1.745A	gag	A*02:01	SLYNTVATL	SLYNTVATL		1
		A*02:01	TLNAWVKVV	TLNAWVKVV		1
		A*02:01	MTSNPPIPV	MTSNPPIPV		1
		A*02:01	VLAEAMSQV	VLAEAMSQV		1
		B*44:02	AEQASQDVKNW	AEQ**CT**QEVKNW	Strong binder	1
	pol	A*02:01	KMIGGIGGFI	KMIGGIGGFI		1
		A*02:01	VLVGPTPVNI	VLVGPTPVNI		1
		A*02:01	ALVEICTEM	ALVEICTEM		1
		A*02:01	VIYQYMDDLYV	VIYQYMDDLYV		1
		A*02:01	ILKEPVHGV	ILKEPVHGV		1
		A*02:01	ALQDSGLEV	ALQDSGLEV		1
		A*32:01	PIQKETWETW	PIQ**R**ETW**D**TW	Non binder	0
		B*44:02	EEMNLPGRW	E**D**M**T**LPGRW	E2D inferred/documented escape	0
		B*44:02	QEEHERYHSNW	Q**DD**HEKYHSNW	Non binder	0
		C*05:01	HTDNGSNF	HTDNG**G**NF	Strong binder	1
	vpr	A*02	AIIRILQQL	AIIRILQQL		1
	rev	B*44:02	EELLKTVRL	EEL**I**KTVRL	L4I diminished response, escape	0
		C*05:01	SAEPVPLQL	**P**AEPVPLQL	Non binder	0
	env	A*02:01	KLTPLCVTL	KLTPLCVTL		1
		A*02:01	TLSQIVTKL	TL**EQVFK**KL	Non Binder	0
		A*02:01	SLLNATDIAV	SLLNATAIAV		1
		A*02:01	RVIEVAQRV	RIIEV**L**QR**A**	Weak binder	0
		A*32:01	RIKQIINMW	RIKQIIN**L**W	Strong binder	1
		B*44:02	AENLWVTVY	**T**E**K**LWVTVY	A1T, N3K documented escape	0
		C*07:04	VYYGVPVWKEA	VYYGVPVWKEA		1
	nef	A*02:01	LEWRFDSTL	L**V**WRFDS**R**L	Non binder	0
		A*02:01	AFHHVAREL	AFHH**M**AREL	Non binder	0
		C*07:04	KRQDILDLWVY	KRQDILDLWVY		1
		C*07:04	RYPLTFGWCY	RYPLTFGWC**F**	Y10F binding unchanged	1
total			29			65.5

P3	region	relevant MHC	expected	observed	impact	WT?
ZFYVE9.745C	gag	A*02:01	SLYNTVATL	SLYNT**L**ATL	Strong binder	1
		A*02:01	TLNAWVKVV	TLNAWVKVV		1
		A*02:01	MTSNPPIPV	MTSNPPIPV		1
		A*02:01	VLAEAMSQV	VLAEAMS**H**V	Strong binder	1
		A*30:02	RSLYNTVATLY	**K**SLYNT**L**ATLY	R1K documented escape	0
	pol	A*02:01	KMIGGIGGFI	KMIGGIGGFI		1
		A*02:01	VLVGPTPVNI	VLVGPTPVNI		1
		A*02:01	ALVEICTEM	ALVEICTEM		1
		A*02:01	VIYQYMDDLYV	VIYQYMDDLYV		1
		A*02:01	ILKEPVHGV	ILKEPVHGV		1
		A*02:01	ALQDSGLEV	ALQDSGLEV		1
		B*44:03	QEEHEKYHSNW	Q**D**EHEKYHSNW	E2D calculated escape	0
		C*04:01	QYDQIPIEI	QYDQIPIEI		1
	vpr	A*02:01	RILQQLLFI	RILQQLLFI		1
	rev	B*44:03	EELLKTVRL	**D**ELLKTVRL	E1D diminished response, documented escape	0
	env	A*02:01	KLTPLCVTL	KLTPLCVTL		1
		A*02:01	TLSQIVTKL	TL**K**QI**AE**KL	Non Binder	0
		A*02:01	SLLNATDIAV	SLLNAT**A**IAV	D7A susceptible variant	1
		A*02:01	RVIEVAQRV	RVIE**IL**QR**A**	Non Binder	0
		B*44:03	AENLWVTVY	E**QQ**LWVTVYY	Non Binder	0
		C*04:01	QFRNKTIVF	QF**EE**NKTIVF	Non Binder	0
		C*04:01	SFNCGGEFF	SFNC**R**GEFF	G5R inferred escape	0
	nef	A*02:01	LEWRFDSTL	LEWRFDS**R**L	Non Binder	0
		A*02:01	AFHHVAREL	AF**R**HVAREL	Non Binder	0
total			24			58.3

P3	region	relevant MHC	expected	observed	impact	WT?	B*57	B*57 WT?
CCND3.d22	gag	A*03:01	KIRLRPGGK	KIRLRPGGK		1		
		A*03:01	RLRPGGKKK	RLRPGGKK**Q**	K9Q diminished response, escape	0		
		B*57:02	AISPRTLNAW	**PL**SPRTLNAW	A1P, I2L documented escape	0	1	
		B*57:02	TSTLQEQIGW	TS**N**LQEQIGW	T3N documented escape, reduced fitness	0	1	
		B*57:02	QATQDVKNW	QATQDVKNW		1	1	1
		B*57:02	KAFSPEVIPMF{S}	KAFSPEVIPMF**{T}**	S+1T literature escape, reduced fitness	0	1	
	pol	A*03:01	AIFQSSMTK	AIFQSSMT**R**	K9R susceptible form	1		
		A*03:01	QIYAGIKVK	QIYAGIKV**R**	K9R diminshed response, documented escape	0		
		B*57:02	STTVKAACWW	S**AA**VKAACWW	Strong binder	1	1	
		B*57:02	PIVLPEKDSW	PI**K**LPEKDSW	Non Binder	0	1	
		B*44:03	EEINLPGRW	E**DM**NLPG**K**W	Non Binder	0		
		B*44:03	QEEHEKYHSNW	Q**D**EHEKYHSNW	E2D calculated escape	0		
	vif	A*29:02	HIVSPRCEY	**RL**VSP**Q**CEY	Weak binder	0		
		B*57:02	ISRKAKGWF	**V**S**K**KAKGW**V**	Non Binder	0	1	
	vpr	B*57:02	AVRHFPRIW	AVRHFPRIW		1	1	1
	tat	A*29:02	NCYCKKCCY	NCYCKKCC**F**	Non Binder	1		
	rev	B*44:03	EELLKTVRL	E**D**LLKTVRL	E2D documented escape,	0		
		B*57:02	LRAVRIIKI	L**KT**VR**L**IK**Q**		0	1	
	env	A*03:01	TVYYGVPVWK	TVYYGVPVWK		1		
		A*03:01	YHQLRDFILIVARTV	Y**RR**LRD**LL**LIVAR**I**V	Non Binder	0		
		A*29:02	SFEPIPIHY	**T**FEPIPIHY	S1T diminished response	0		
		A*29:02	SFNCGGEFFY	SFNC**A**GEFFY	Strong binder	1		
		B*44:03	AENLWVTVY	**K**E**Q**LWVTVY	Strong binder	1		
	nef	A*03:01	QVPLRPMTYK{AA}	QVPLRPMTYK{**G**A}	A+1G inferred escape	0		
		A*03:01	ALDLSHFLR	A**M**DL**G**HFL**K**	Strong binder	1		
		B*57:02	KAAFDLSFF	K**G**A**M**DL**GH**F	T2G diminished response	0	1	
		B*57:02	HTQGYFPDW	HTQGYFPDW		1	1	1
total			27			40.7	9	22.2

Supplementary Table S4. Analysis of CTL escape

Primers used for single genome sequencing (SGS) PCR

Assay	Primer name	PCR reaction	Primer type	Primer sequence	reference
**u5-gag**	u5gagSGS_fo	outer	forward	GTARCTAGAGATCCCTCAGAC	Simonetti et al. (33301425)
	u5gagSGS_ro		reverse	TGACATGCTGTCATCATYTCYTC	
	u5gagSGS_fn	nested	forward	AAATCTCTAGCAGTGGCGCC	
	u5gagSGS_rn		reverse	CATCATTTCTTCTARTGTAGCTSCT	
**P6-RT**	1840+	outer	forward	GATGACAGCATGTCAGGGAG	Van Zyl et al. (28891813)
	3500-		reverse	CTATYAAGTCTTTTGATGGGTCATAA	
	1870+	nested	forward	GAGTGTTGGCTGAGGCAATGAG	
	3410-		reverse	CAGTTAGTGGTACTATGTCTGTTAGTGCTT	
**env**	envSGS_fo	outer	forward	GCCAGTAGTRTCAACYGAA	Simonetti et al. (33301425)
	envSGS_ro		reverse	GCARATGAGTTTTCYAGAGCA	
	envSGS_fn	nested	forward	CTGCTAAATGGCAGTCTAGC	
	envSGS_rn		reverse	TTGCCTGGAGCTGYTTRATGC	

Primers used for cDNA synthesis from plasma or cell-associated RNA

Assay	Primer name	Reaction	Primer type	Primer sequence	reference
**u5-gag**	u5gagSGS_ro	cDNA	reverse	AAATCTCTAGCAGTGGCGCC	
**P6-RT**	3500-	cDNA	reverse	CTATYAAGTCTTTTGATGGGTCATAA	
**env**	Env.B3	cDNA	reverse	TTGCTACTTGTGATTGCTCCATGT	Bertagnolli et al. (33239444)

Primers used for Sanger sequencing

Assay	Primer name	PCR reaction	Primer type	Primer sequence	reference
**u5-gag**	u5gagSGS_fn	Sanger	forward	AAATCTCTAGCAGTGGCGCC	Simonetti et al. (33301425)
	INT3A2	Sanger	reverse	AGCTTCCTCATTGATGGTCTCTTT	
	u5gagSGS_rn	Sanger	reverse	CATCATTTCTTCTARTGTAGCTSCT	
**P6-RT**	1870+	Sanger	forward	GAGTGTTGGCTGAGGCAATGAG	Van Zyl et al. (28891813)
	3410-	Sanger	reverse	CAGTTAGTGGTACTATGTCTGTTAGTGCTT	
**env**	envSGS_fn	Sanger	forward	CTGCTAAATGGCAGTCTAGC	Simonetti et al. (33301425)
	envSGS_rn	Sanger	reverse	TTGCCTGGAGCTGYTTRATGC	

Primers and probes used for to confirm integration site

Assay	Primer name	PCR reaction	Primer type	Primer sequence	reference
**ADK**	ADK_FO	outer	forward	AGCCGTATCTACGGGTTACTGG	
	ADK_FN	nested	forward	GTTACTGGAAGGGCTAATTTACTC	
**AAK1**	AAK1_FO	outer	forward	GAGAAATCCAGTAACCAATCATGAC	
	AAK1_FN	nested	forward	CCTTGCAAGGACTGACTTTTGAG	
**DNAJB14**	DNAJB14_FO	outer	forward	TACTTTGTGCTATACCCATATAATCAAG	
	DNAJB14_FN	nested	forward	TTGGTATTCATTGGAAGGGCTAATTC	
**RRM1**	RRM1_FO	outer	forward	TATCAATAATCTCTTCACTGCCTTG	
	RRM1_FN	nested	forward	GGATTCTATCTATTGACTAGGTGT	
**ZFYVE9**	ZFYVE9_FO	outer	forward	AAACATGCCAATGCCTCTGGC	
	ZFYVE9_FN	nested	forward	CTAAACACTTGGAAAAATGATTGAG	
**CCND3**	CCND3_FO	outer	forward	CAAAGAAAAGATGGAGTTCCC	
	CCND3_FN	nested	forward	GAAGGCCTGGAGAAAACTAGC	
**CCND3**	CCND3_RO	outer	reverse	AAGACGTCTGTTTATTATTCGCAAAG	
	CCND3_RN	nested	reverse	CTCTGGTTTGTTAAGCTTTGCCA	

Primers and probes used for HIV RNA quantification

Assay	Primer name	PCR reaction	Primer type	Primer sequence	reference
**U5-R**	RU5-F	digital PCR	forward	CTTAAGCCTCAATAAAGCTTGCC	Anderson et al. (30253074)
	RU5-Probe	digital PCR	probe	5fam/AGTAGTGTG/zen/TGCCCGTCTG/3iabk	
	RU5-R	digital PCR	reverse	GGATCTCTAGTTACCAGAGTC	
**ADK.d22 deletion**	ADK_d22_FWD	digital PCR	forward	CAGGACTCGGCTTGCTGAG	
	ADK_d22_PRB	digital PCR	probe	5fam/CGCACGGCA/zen/AGAGTACGCCATAAA/3iabk	
	ADK_d22_REV	digital PCR	reverse	GCACCCATCTCTCTCCTTCTAGC	
**DNAJB14.d21 deletion**	DNAJB_d21_FWD	digital PCR	forward	CGAAAGGGAAACCAGAGGAG	
	DNAJB_d21_PRB	digital PCR	probe	5fam/CTCCGCTAG/zen/TCAAACGCCGC/3iabk	
	DNAJB_d21_REV	digital PCR	reverse	CGCTTAATACCGACGCTCTC	
**ADK.d22 read through**	ADK-U3_FWD	digital PCR	forward	AGGCATTGTGCTAAGTGAGAA	
	ADK-U3_PRB	digital PCR	probe	5hex/CTACGGGTT/zen/ACTGGAAGGGCTAATTTAC/3iabk	
	ADK-U3_REV	digital PCR	reverse	GTGGTGAACCCACAGATCAA	
**DNAJB14.d21 read through**	DNAJB14-U3_FWD	digital PCR	forward	GTGGCCAGAGTAAATTTCTATTCAT	
	DNAJB14-U3_PRB	digital PCR	probe	5hex/ATTTGGTA/zen/TTCATTGGAAGGGCTAATTC/3iabk	
	DNAJB14-U3_REV	digital PCR	reverse	GTGGTAGACCCACAGATCAAG	

Primers used for single genome sequencing (SGS) of spliced HIV transcripts

Assay	Primer name	PCR reaction	Primer type	Primer sequence	reference
**1.8Kb**	625_FO	outer	forward	ACTTGAAAGTGAAAGTAGAACCAG	Adapted from Emery et al. (28077653)
	1.8_REV		reverse	CAGTTCGGGATTGGGAGGTGGGTTGC	
	678_FN	semi_nested	forward	GAGCTCTCTCGACGCAGGAC	
	1.8_REV		reverse	CAGTTCGGGATTGGGAGGTGGGTTGC	
**4.0Kb**	625_FO	outer	forward	ACTTGAAAGTGAAAGTAGAACCAG	
	4_REV		reverse	GTACTATAGGTTGCATTACATGTACTACTTAC	
	678_FN	semi_nested	forward	GAGCTCTCTCGACGCAGGAC	
	4_REV		reverse	GTACTATAGGTTGCATTACATGTACTACTTAC	

Primers and probes used for U5PBS quantification (late RT product)

Assay	Primer name	PCR reaction	Primer type	Primer sequence	reference
**U5-PBS**	U5PBS_FWD	digital PCR	forward	CTGTTGTGTGACTCTGGTAACT	
	U5PBS_PRB	digital PCR	probe	5fam/CAGTGGCGC/zen/CCGAACAGGGACTTG/3iabk	
	U5PBS_REV	digital PCR	reverse	AGTCCTGCGTCGAGAGAT	

Primers and probes used for site directed mutagenesis

Assay	Primer name	PCR reaction	Primer type	Primer sequence	reference
**NL4-3_d22**	d22_SDM_F	Q5 PCR	forward	GTACGCCAAAAATTTTGACTAGCGG	
	d22_SDM_R	Q5 PCR	reverse	TCTTGCCGTGCGCGCTTC	
**NL4-3_d21**	d21_SDM_F	Q5 PCR	forward	TTTGACTAGCGGAGGCTAGAAGGAGAGAG	
	d21_SDM_R	Q5 PCR	reverse	CGCCGCCCCTCGCCTCTT	

Primers and probes used for HIV-1 RNA quantification from transfected cells

Assay	Primer name	PCR reaction	Primer type	Primer sequence	reference
**PolyA**	Freadth-2	digital PCR	forward	GCCCTCAGATGCTRCATATAA	Yukl et al. (29491188)
	Preadth-1	digital PCR	probe	5fam/TGCCTGTTA/zen/CTGGGTCTCTCGGTTAG/3iabk	
	5T25	digital PCR	reverse	TTTTTTTTTTTTTTTTTTTTTTTTTGAAG	
**4kb-class**	4kb_FWD	digital PCR	forward	TGAAAGCGAAAGTAAAGCCAGAG	
	4kb_PRB	digital PCR	probe	5fam/AGATCTCTC/zen/GACGCAGGACTCGG/3iabk	
	4kb_REV	digital PCR	reverse	GTTGCATTACATGTACTACTTACTGC	
**tat/rev**	mf1	digital PCR	forward	CTTAGGCATCTCCTATGGCAGGAA	Yukl et al. (29491188)
	Mf226mod	digital PCR	probe	5fam/ACCCGACAGGCC/3iabk	
	mf83	digital PCR	reverse	GGATCTGTCTCTGTCTCTCTCTCCACC	

Primers and probes used for duplex LTR and integration site quantification

Assay	Primer name	PCR reaction	Primer type	Primer sequence	reference
**U5-R**	RU5-F	digital PCR	forward	CTTAAGCCTCAATAAAGCTTGCC	Anderson et al. (30253074)
	RU5-Probe	digital PCR	probe	5fam/AGTAGTGTG/zen/TGCCCGTCTG/3iabk	
	RU5-R	digital PCR	reverse	GGATCTCTAGTTACCAGAGTC	
**ADK-U3**	ADK-U3_FWD	digital PCR	forward	AGGCATTGTGCTAAGTGAGAA	
	ADK-U3_PRB	digital PCR	probe	5hex/CTACGGGTT/zen/ACTGGAAGGGCTAATTTAC/3iabk	
	ADK-U3_REV	digital PCR	reverse	GTGGTGAACCCACAGATCAA	
**DNAJB14-U3**	DNAJB14-U3_FWD	digital PCR	forward	GTGGCCAGAGTAAATTTCTATTCAT	
	DNAJB14-U3_PRB	digital PCR	probe	5hex/ATTTGGTA/zen/TTCATTGGAAGGGCTAATTC/3iabk	
	DNAJB14-U3_REV	digital PCR	reverse	GTGGTAGACCCACAGATCAAG	
**ZFYVE9-U3**	ZFY-U3_FWD	digital PCR	forward	AAACATGCCAATGCCTCTG	
	ZFY-U3_PRB	digital PCR	probe	5hex/CGGCCGAGG/zen/TGGCTCTGGAAGG/3iabk	
	ZFY-U3_REV	digital PCR	reverse	GTTATCCCAATCAGGGAAGAAAC	
**CCND3-U3**	CCND3-U3_FWD	digital PCR	forward	GACTGGAAAGAAGGAAGAGAAGG	
	CCND3-U3_PRB	digital PCR	probe	5hex/TGAGGGCTT/zen/GTAACTGGAAGGGCTAATTTAC/3iabk	
	CCND3-U3_REV	digital PCR	reverse	AGACCCACAGATCAAGGATTTC	

Supplementary Table S5. Oligos used in this study. Numbers in parenthesis represent reference PMID.
