# Supplementary material for: Clonally expanded HIV-1 proviruses with 5′-leader defects can give rise to nonsuppressible residual viremia
Source: J Clin Invest. 2023 Mar 15;133(6):e165245. doi: 10.1172/JCI165245 (PMC10014112; doi:10.1172/JCI165245)

Original blot images for Figure 3B.

Top panel, WT NL4-3

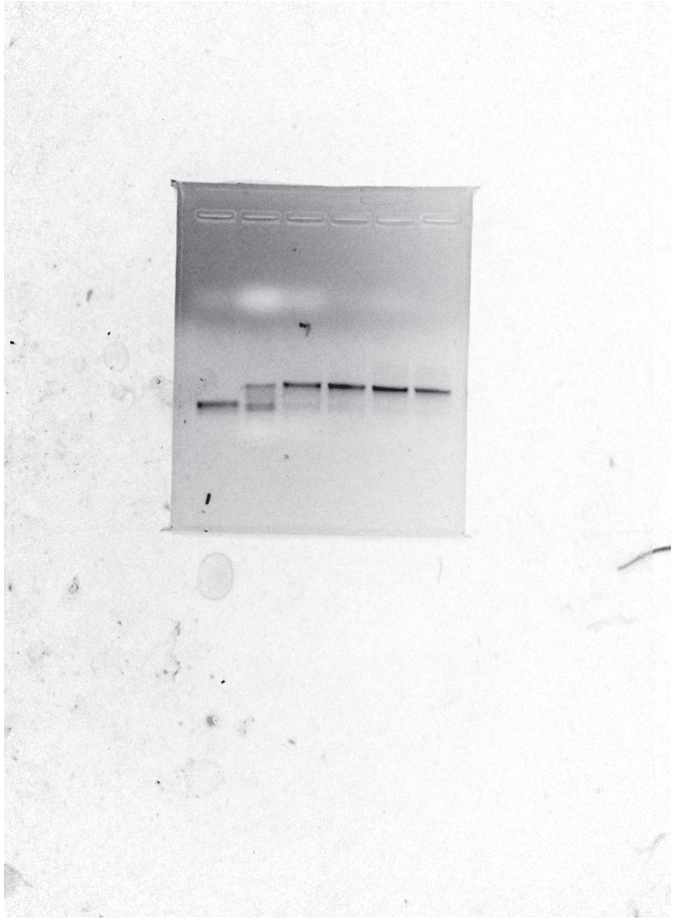

Middle panel, D22 NL4-3

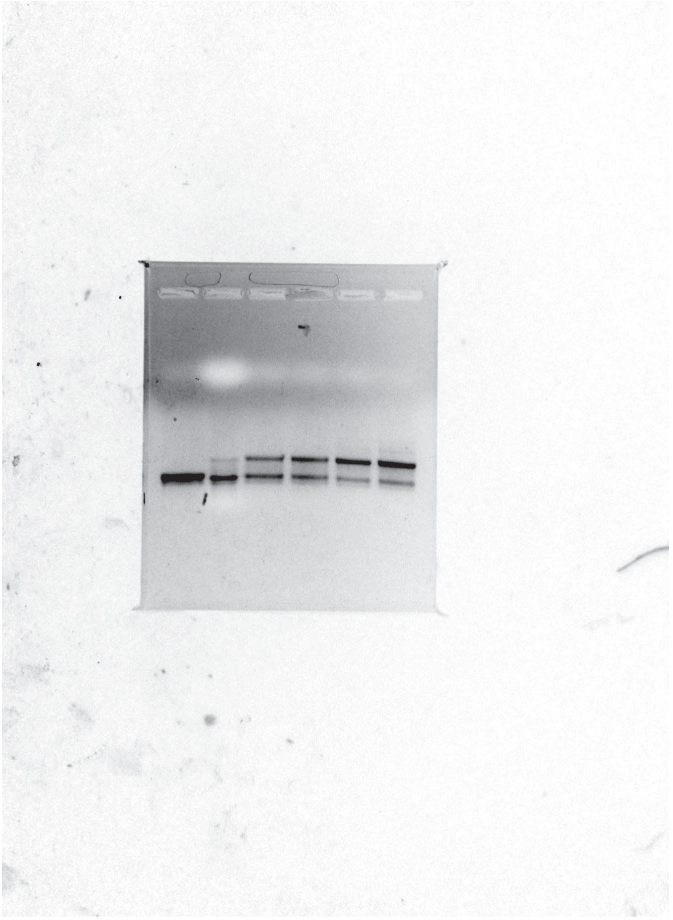

Bottom panel, D21 NL4-3

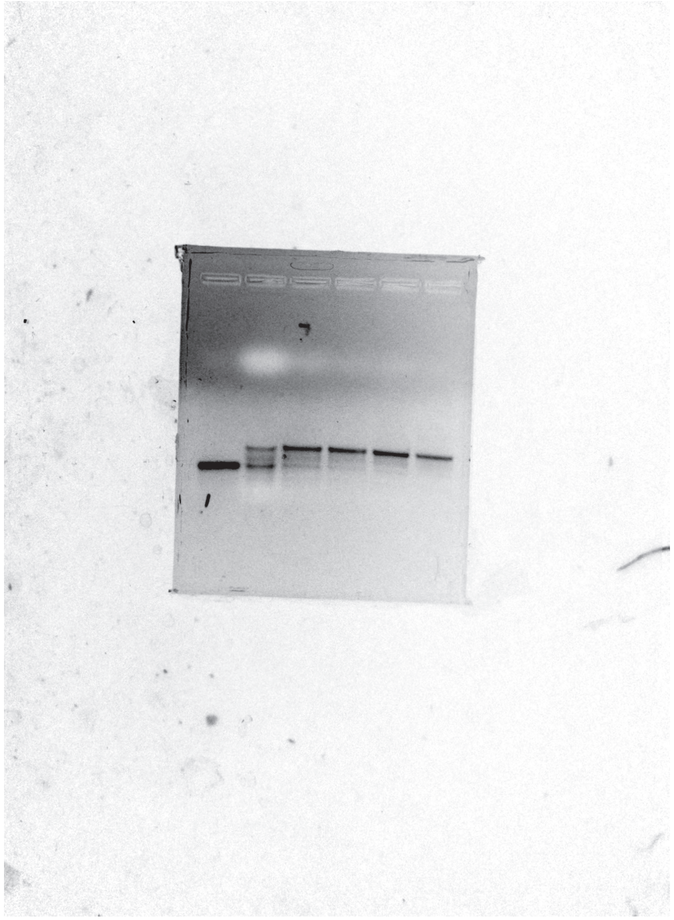

Original western blot images used for Figure 5.

Membrane with mass ladder

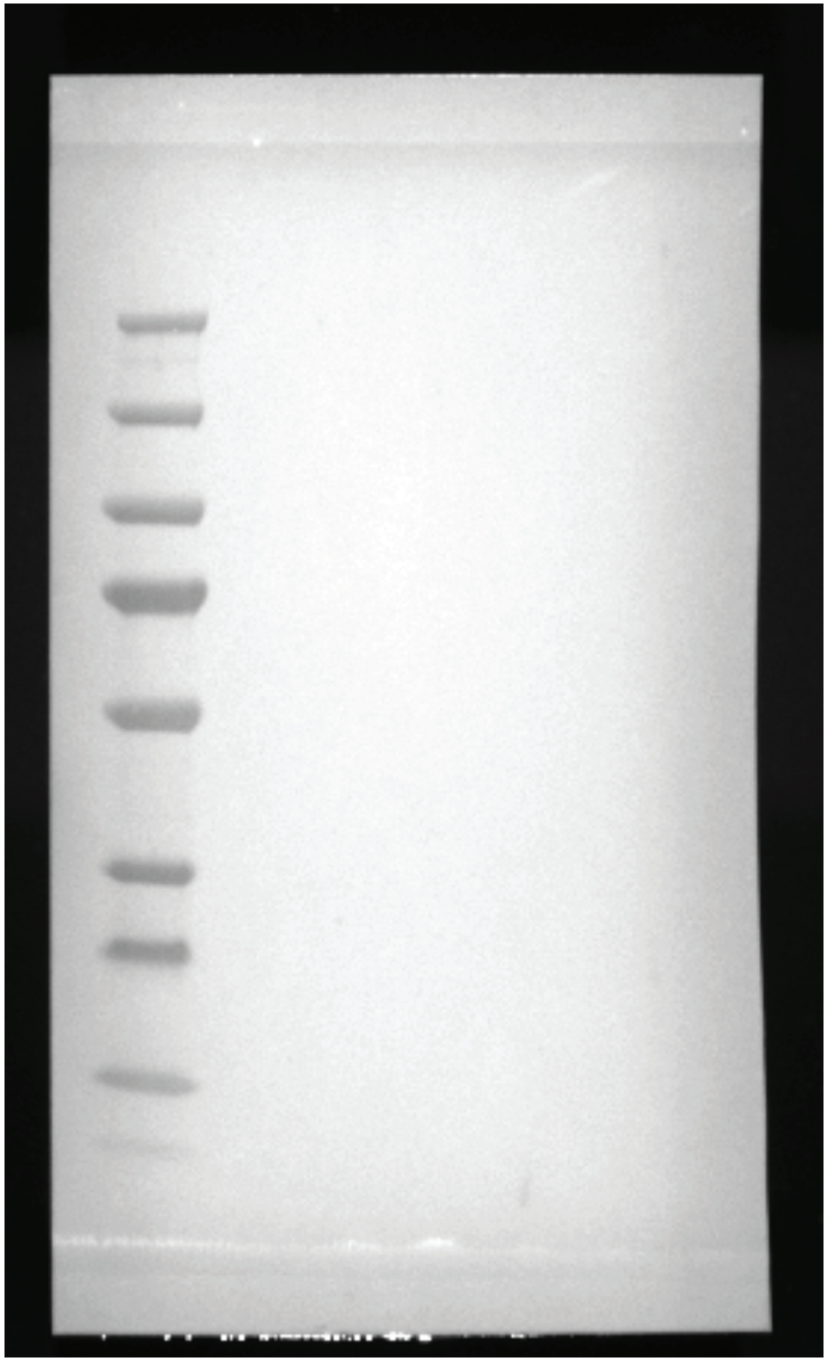

Chemi luminescent signal with secondary Ab for p24

NL4-3 WT | NL4-3 d22 | NL4-3 d21

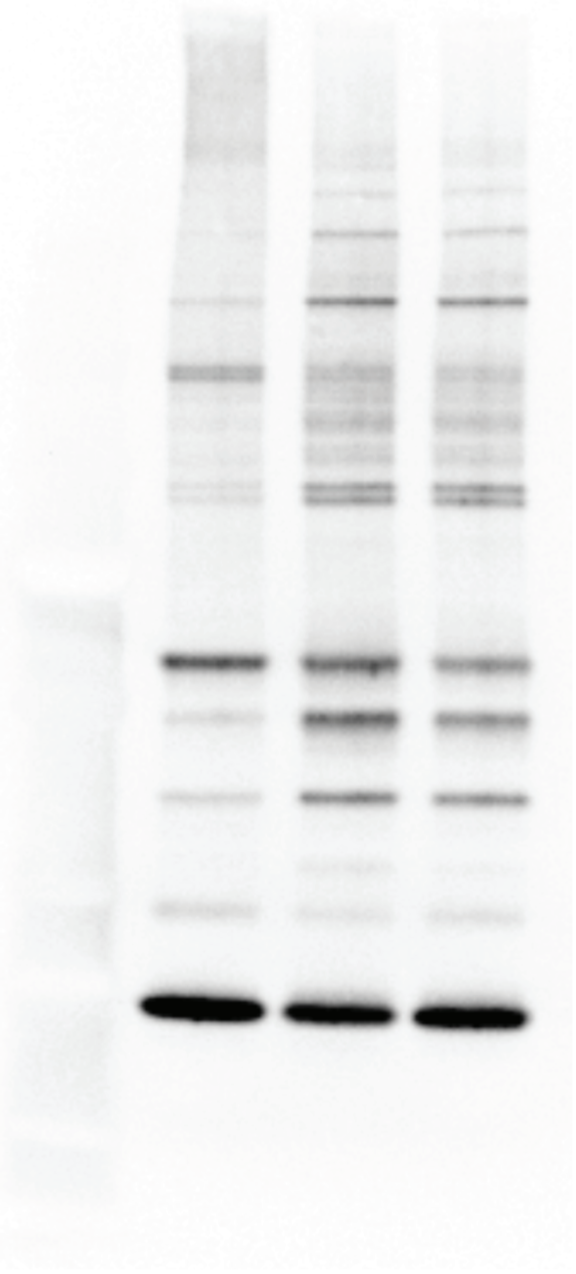

Chemi luminescent signal with secondary Ab for gp41

NL4-3 WT | NL4-3 d22 | NL4-3 d21

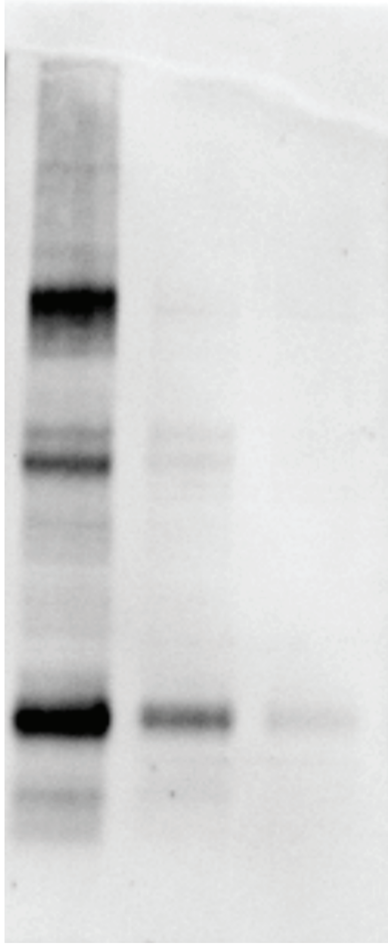

Supplement: Supplemental uncut gels [file jci-133-165245-s077.pdf]
